# The efficacy of prevention for colon cancer based on the microbiota therapy and the antitumor mechanisms with intervention of dietary *Lactobacillus*

**DOI:** 10.1128/spectrum.00189-23

**Published:** 2023-09-01

**Authors:** Fuqiang Xu, Qiaoqiao Li, Shuyang Wang, Miaoyin Dong, Guoqing Xiao, Jin Bai, Junkai Wang, Xisi Sun

**Affiliations:** 1 Institute of Modern Physics, Chinese Academy of Sciences, Lanzhou, Gansu, China; 2 College of Nuclear Science and Technology, University of Chinese Academy of Sciences, Beijing, China; Brigham Young University, Provo, Utah, USA

**Keywords:** *Lactobacillus*, colon cancer, gut microbiota, serine, sphingosine 1-phosphate signaling

## Abstract

Gut microbiota and their secreted metabolites have an influence on the initiation and progression of colon cancer. Probiotics are extensively perceived as a potential microbiota-modulation strategy to promote the health of the host, while the effectiveness of preventing colon cancer based on microbiota therapy has not been confirmed, and antitumor mechanisms influenced by microbiota and their metabolites with the intervention of probiotics remain to be further investigated. *In vitro*, *Lactobacillus* (JY300-8 and JMR-01) significantly inhibited the proliferation of CT26, HT29, and HCT116 cells. Moreover, we studied the prevention and therapy efficiency of *Lactobacillus* and its underlying antitumor mechanism through the alteration of gut microbiota and their metabolites regulated by *Lactobacillus* in colon cancer models in mice. We demonstrated that the pre-administration of *Lactobacillus* (JY300-8 and JMR-01) for 20 days before establishing tumor models resulted in an 86.21% reduction in tumor formation rate compared to tumor control group. Subsequently, continuous oral administration of living *Lactobacillus* significantly suppresses tumor growth, and tumor volumes decrease by 65.2%. Microbiome and metabolome analyses reveal that *Lactobacillus* suppresses colonic tumorigenesis and progression through the modulation of gut microbiota homeostasis and metabolites, including the down-regulation of secondary bile acids, sphingosine 1-phosphate (S1P), and pyrimidine metabolism, as well as the production of anticarcinogenic compounds in tumor-bearing mice. Additionally, metabolome analyses of *Lactobacillus* (JY300-8 and JMR-01) indicate that living *Lactobacillus* could reduce the relative abundance of alanine and L-serine to suppress tumor progression by regulating the tumor microenvironment, including down-regulation of pyrimidine metabolism and S1P signaling in cancer. These findings provide a potential prevention strategy and therapeutic target for colon cancer through the intervention of dietary *Lactobacillus*.

**IMPORTANCE:**

The modulation of gut microbiota and metabolites has a significant influence on the progression of colon cancer. Our research indicated that the intervention of probiotics is a potentially feasible strategy for preventing colon cancer. We have also revealed the underlying antitumor mechanism through the alteration of gut microbiota and their metabolites, which could lead to broader biomedical impacts on the prevention and therapy of colon cancer with microbiota-based therapy regulated by probiotics.

## INTRODUCTION

Colorectal cancer (CRC) is the second leading cause of cancer-related deaths, with nearly 1.0 million deaths occurring in 2020 (1). The polypoid adenomas in the colon and rectum can develop CRCs through a multistep mechanism, including the adenoma-carcinoma process (2). Most patients are diagnosed at the middle and advanced stages due to the longer development of malignancies in the colon and rectum, as well as higher concealment compared to other cancers. This results in a shorter treatment window and higher mortality rate (3, 4). Therefore, efficient and safe prevention strategies for colon cancer in early stages and the development of therapeutic target for a new treatment strategy are priorities for the control of colon cancer.

One-fifth of human malignancies are associated with gut microbiota (5). Accumulating pieces of evidence show that gut microbiota is closely related to digestive diseases of occurrence and the maintenance of health, especially colon cancer (6, 7). Therefore, the regulation of gut microbiota has been considered as a promising strategy for preventing and treating colon cancer (8, 9). Previously, phage-based intervention of gut microbiota and fecal microbiota transplantation (FMT) were shown to alter the gut microbiota of the patients and remodel the homeostasis of intestinal microbiota, resulting in positive therapeutic effects (10, 11). However, the safety and operation standardization remain challenges for patients with colon cancer, which limits the development of cancer therapy based on modulation of microbiota through phage-based intervention and FMT. Probiotics, as innovative functional foods that modulate the microbiota to enhance host health and inhibit tumor carcinogenesis, have attracted extensive interest. Importantly, the safety and complete standardization of probiotics have been confirmed (12). Recent studies revealed potential positive effects of probiotics to colon cancer therapy (13, 14). Similarly, our previous results showed that *Lactobacillus casei* significantly inhibited tumor progression in animal models of colon cancer (15). However, the relationship between colon cancer and the microbes is complex. The mechanism by which the microbes and the metabolites contribute to carcinogenesis, as well as the antitumor effects influenced by microbiota and their metabolites with the intervention of probiotics, remains unclear.

Observing the roles of microbes, the microbiota and their metabolites in colon cancer models in mice are crucial for early prevention and treatment of colon cancer. Fecal microbial diversity is a useful tool for studying an altered gut microbiome. Correspondingly, the metabolites, such as short-chain fatty acids (SCFAs), hydrogen sulfide, acetaldehyde, and secondary bile acids, are involved in the initiation and/or progression of colorectal cancer (16). Therefore, the comprehensive microbiome and metabolomic analyses might provide an alternative approach to understand the occurrence and development of colon cancer, as well as the associated alteration in the gut microbiota and their secreted metabolites with the intervention of probiotics.

The aim of this study is to investigate the preventive and therapeutic efficacy of *Lactobacillus* (JY300-8 and JMR-01) for colon cancer, as well as the underlying antitumor mechanism through modulation of gut microbiota and their metabolites regulated by *Lactobacillus*. The preventive and therapeutic efficacy is assessed by orally administering *Lactobacillus* for 20 days in advance in subcutaneous tumor models. Moreover, fecal microbiota and their metabolic profiles are analyzed to obtain evidence of antitumor effects exerted by fecal microorganisms and metabolites. The nontargeted metabolome analysis for probiotics is employed to further identify anticolon cancer metabolites produced by living bacteria and inactivated bacteria.

## RESULTS

### The antiproliferation ability of *Lactobacillus* against colon cancer cells *in vitro*

**Fig 1:**
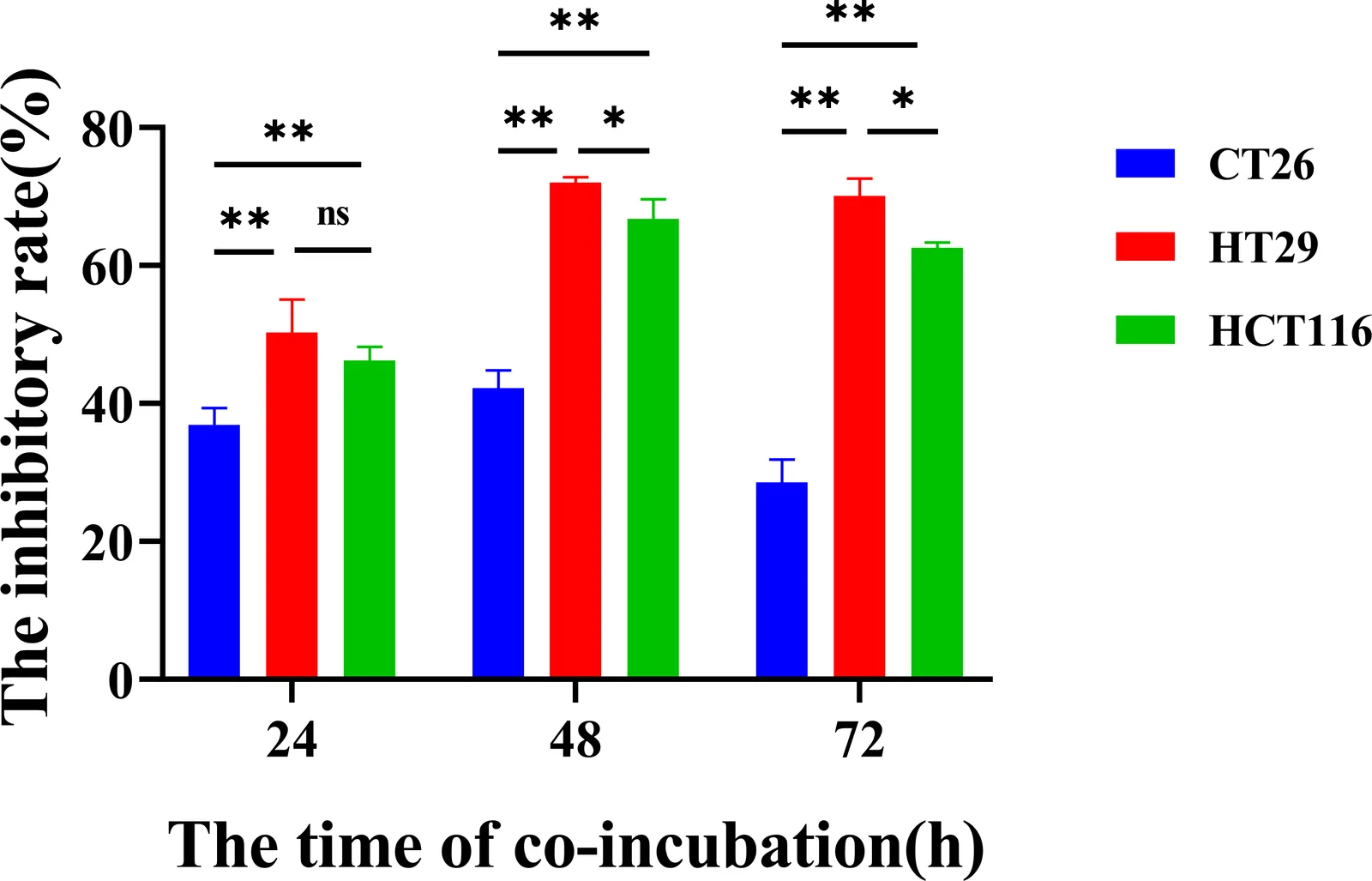
Antiproliferative effects of probiotic (JY300-8 and JMR-01) on colon cancer cells (ns, not significant, *P < 0.05, **P < 0.01).

The probiotics (*L. casei* JY300-8 and *L. reuteri* JMR-01) significantly inhibited the proliferation of colon cancer cells, including murine colon cancer cell lines CT26 cell and human colorectal adenocarcinoma cell lines (HT29 and HCT116 cells) (Fig. 1).

The inhibitory activity of JY300-8 and JMR-01 against colon cancer cells gradually increased with the duration of co-incubation, reaching its highest level after 48 h. Particularly, the probiotics significantly improved the inhibitory effect on human colorectal adenocarcinoma cell lines (HT29 and HCT116 cells) in comparison to the CT26 cell. After 48 h of co-cultivation at a probiotic to cell ratio of 1:1, the probiotics (JY300-8 and JMR-01) markedly inhibited the proliferation of HT29 and HCT116 cells, with inhibitory rates of 72.02% and 66.76%, respectively. Compared to the inhibitory rate of probiotics on CT26 cells, there was a significant increase in the inhibitory rate for HT29 and HCT116 cells by 70.46% and 58.01%, respectively. The results suggest that probiotic (JY300-8 and JMR-01) is effective in inhibiting the proliferation of CT26, HT29, and HCT116 cells, which lays a foundation that JY300-8 and JMR-01 could constrain colon cancer *in vivo*.

### Antitumor efficacy of *Lactobacillus* in subcutaneous colon cancer models in mice

**Fig 2:**
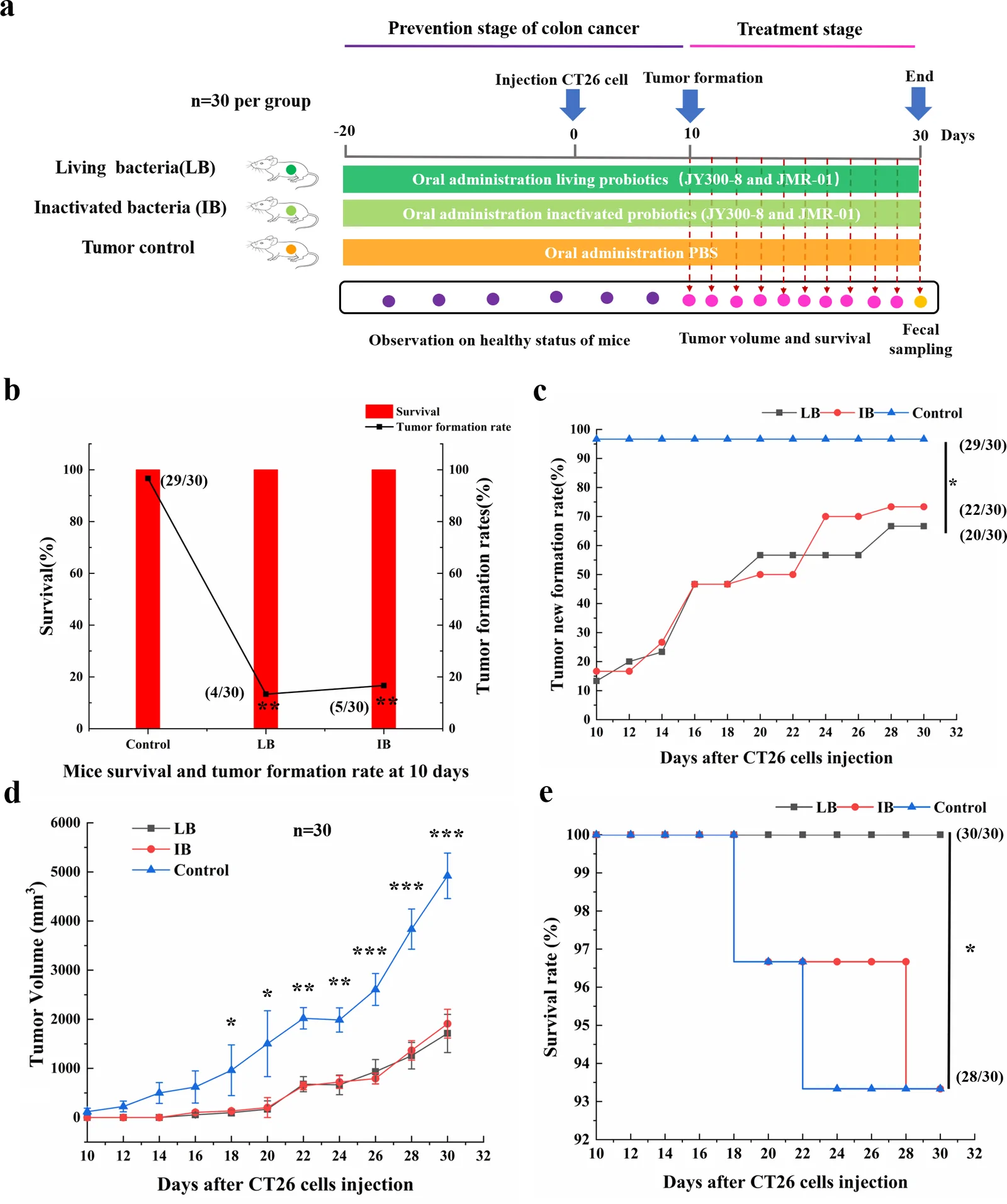
*Lactobacillus* suppress the occurrence and progression of colon cancer induced by CT26 cells in mice. (a) The experimental scheme for tumor prevention and treatment by oral administration of JY300-8 and JMR-01. Mice (*n* = 30 per group) were administrated with LB, IB, and PBS for 20 days in advance. Subsequently, they were injected with CT26 cells subcutaneously, followed by schemed treatment for 10 days until the mean volume reached 100 mm^3^ in tumor control group. The survival and tumor formation rates were then assessed. Subsequently, schedules indicating the measurement of tumor volume, survival, new formation rate of tumor, and fecal sampling are shown with arrows. (b) The survival and tumor formation rates in three groups at 10 days when mice in control group were successfully established as colon cancer models, **P < 0.01 for control vs LB, control vs IB. (c) The new formation rates of tumor were assessed during the experiment. (d) The kinetics of tumor growth in tumor-bearing BALB/c mice (means ± SEM), *P < 0.05, **P < 0.01, ***P < 0.001 for control vs LB/IB. (e) The survival of tumor-bearing mice in all groups.

To determine whether *Lactobacillus* exerted prevention and treatment effect on colon cancer *in vivo*, we studied the antitumor efficacy of JY300-8 and JMR-01 in CT26 cells-induced colon cancer models in mice (Fig. 2). The experimental scheme for tumor prevention and treatment by oral administration of *Lactobacillus* is shown in Fig. 2a. The tumor formation rate in the control group reached 96.67% at the tenth day after injection of CT26 cells, with only one mouse not developing colon cancer. In contrast, the tumor formation rate is merely 13.33% and 16.67% when orally administrated with living bacteria (JY300-8 and JMR-01) and inactivated bacteria, respectively (Fig. 2b). Compared to the tumor control group, the tumor formation rate in the living bacteria (LB) group and the inactivated bacteria (IB) group was reduced by 86.21% and 82.27% (*P* < 0.01), respectively. However, there was no statistical difference between LB group and IB group. Therefore, our results suggest that oral administration of both living bacteria and inactivated bacteria significantly suppress colonic tumorigenesis induced by CT26 cell in mice. During the treatment stage of colon cancer in mice, tumors with a volume less than 100 mm^3^ gradually grew and formed an obvious tumor (Fig. 2c). At the end of the experiment, a remarkable 33.33% of mice in the LB group did not exhibit any discernible tumors, which is significantly higher than that observed in the control group (3.33%) (*P* < 0.05). These results provide compelling evidence that oral administration of living *Lactobacillus* can effectively mitigate the growth of colon cancer.

Correspondingly, the tumor volume of tumor-bearing mice is dramatically reduced when administrated with living bacteria and inactivated bacteria compared to the tumor control group (Fig. 2d). The mean tumor volume is 4920.63 ± 462.40 mm^3^ in tumor control group, while it is 1712.45 ± 388.95 mm^3^ and 1909.95 ± 292.90 mm^3^ in living bacteria and inactivated bacteria groups at 30 days after CT26 cell injection, respectively. The tumor volume decreases by 65.2% and 61.19% in LB and IB in comparison to the tumor control group (*P* < 0.001), while there is no significant difference between LB and IB group. Therefore, oral administration of both living bacteria and inactivated bacteria remarkably inhibits the progression of colon cancer induced by CT26 cell in mice. Moreover, the survival rate of tumor-bearing mice significantly decreases to 93.33% in both tumor control group and IB group at 30 days after CT26 cell injection; however, there is 100% survival rate when living bacteria (JY300-8 and JMR-01) are orally administrated (Fig. 2e). The results suggest that administration of living bacteria (JY300-8 and JMR-01) has a potential advantage of prolonging the survival of tumor-bearing mice compared to inactivated bacteria. In summary, our results demonstrate that the living probiotics (JY300-8 and JMR-01) significantly suppress tumorigenesis and the progression of colon cancer, thereby prolonging the survival of tumor-bearing mice.

### *Lactobacillus* enhances antitumor responsiveness by modulating the gut microbiota in mice

**Fig 3:**
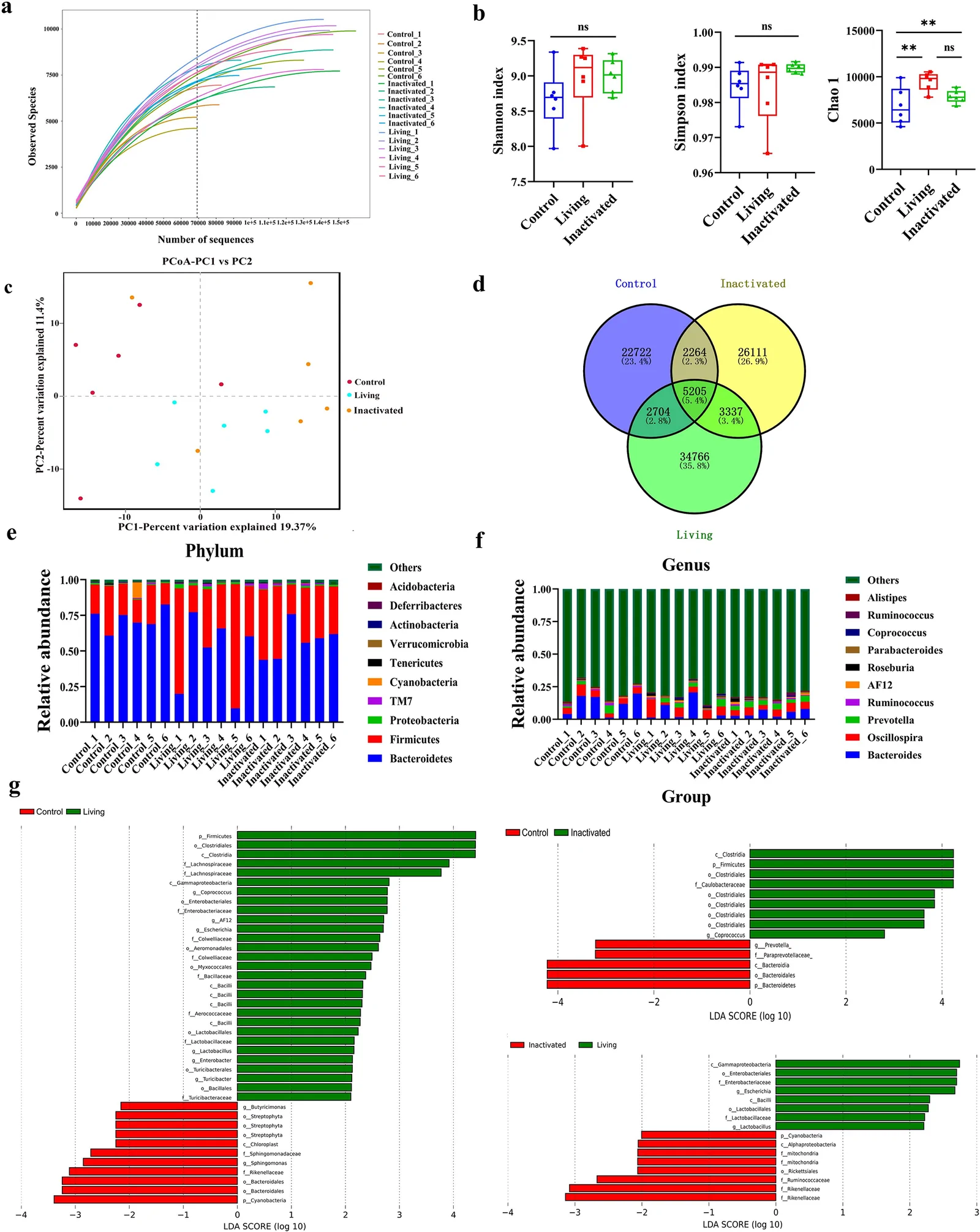
The analysis of gut microbiota modulated by *Lactobacillus* in tumor-bearing mice. (a) The rarefaction curve of microbial communities in fecal samples. (b) Changes in alpha diversity indices of microbial communities in fecal samples, including Shannon, Simpson, and Chao 1 indices, were observed. (c) Principal coordinate analysis based on Bray–Curtis dissimilarity. Each dot represents a single sample and each group is shown in a different color. (d) Venn diagram analysis of OTU overlaps between different groups of microorganisms. (e) Relative abundance at the phylum level. (f) Relative abundance at the genus level. (g) Linear discriminant analysis (LDA) effect size of fecal samples after various treatments. The red or green columns in the histogram represent the microbial groups that played a vital role in their group. The figure only shows microbiota whose LDA score is greater than 2. The length of the bar chart represents the size of the LDA value.

To further investigate the effects of *Lactobacillus* on gut microbiota in colon cancer models in mice and assess the interaction between altered microbiota and colon cancer progression, we performed MiSeq sequencing analysis of the V3-V4 region of 16S ribosomal RNA gene from fecal samples. The structure and abundance of microbiota in three groups are shown in Fig. 3. According to the rarefaction curve (Fig. 3a), the quality of sequencing data in samples is sufficient to perform the analysis of the diversity and abundance of gut microbiota. The diversity of microbial flora (Shannon index and Simpson index) did not show significant differences among tumor control (CK), living bacteria, and inactivated bacteria groups (Fig. 3b). The abundance of microbial flora, both in living and inactivated bacteria groups, was significantly higher than that of the tumor control group (*P* < 0.001). However, there was no significant difference between living and inactivated bacteria groups (Fig. 3b). Additionally, the results of principle-coordinates analysis (PCoA) indicate that there are significant differences in phylogenetic community structures between the tumor control group and other groups. However, the PCoA plots also suggest that the living bacteria and inactivated bacteria groups are not apparently dispersed at 30 days (Fig. 3c). It demonstrates that administration of *Lactobacillus* significantly alters the composition of gut microbiota in tumor-bearing mice. Venn analysis also shows that the structure of the intestinal flora can be affected by oral administration of living bacteria (Fig. 3d). Distribution and abundance of microbial taxa at the phylum level in three groups at 30 days are examined as shown in Fig. 3e. The results indicate that the dominant phyla in the gut microbiota are *Bacteroidetes*, *Firmicutes*, *Proteobacteria,* and *TM7* (with a relative abundance >0.5%). At the genus level, the fluctuation of relative abundance depends on the different treatment. The dominant genera in gut microbiota are *Bacteroides, Oscillospira, Prevotella, Ruminococcus, AF12, Roseburia, Parabacteroides, Coprococcus,* and *Ruminococcus* (with a relative abundance >0.5%) (Fig. 3f). Compared to the control group, there was a significant increase in microbes, including *Oscillospira*, *Prevotella*, AF12, *Roseburia,* and *Coprococcus* in the living bacteria group. However, *Bacteroides*, *Ruminococcus,* and *Parabacteroides* decreased. These results show that the gut microbiota with the capacity of producing antitumor compounds are enriched in living bacteria group. Similarly, *Prevotella*, *AF12*, *Roseburia, Coprococcus,* and *Ruminococcus* increased, while *Bacteroides* and *Ruminococcus* decreased in the inactivated bacteria group in comparison to the tumor control group. Thus, our data demonstrate that both living bacteria and inactivated bacteria contribute to alleviating dysbiosis induced by tumor by modulating gut microbiota structure, increasing alpha and beta diversity, and improving the proportion of potentially beneficial microbes.

Furthermore, we employed linear discriminant analysis (LDA) and effect size (LEfSe) methods to identify the bacteria that are most likely associated with the preventive and therapeutic efficacy of colon cancer. The lists of taxonomic clades were ranked according to their effect size, as shown in Fig. 3g. The results indicate that *Coprococcus, AF12, Lactobacillus, Enterobacter,* and *Turicibacter* are more discriminative at genus level in living bacteria compared to the control group. Similarly, *Coprococcus* is more differential in inactivated bacteria. Notably, *Lactobacillus* is enriched in living bacteria compared to inactivated bacteria. Additionally, *Butyricimonas*, *Sphingomonas,* and *Prevotella* are the most discriminative among tumor control than others.

### The analysis of untargeted metabolites associated with antitumor efficacy regulated by *Lactobacillus*

The untargeted metabolomics technology is used to obtain the complete metabolic profiles of gut microbiota in colon cancer mice with different treatments. Overall, we obtained metabolites from identification level 1 to level 4, thereby enhancing the reliability of our compound identification results. Additionally, the sum of strength values represents peak intensity, which refers to the area of the peak in the mass spectrum and serves as an indicator of signal strength. Based on MS-generated raw data, the ratio between the sum of strength values that the relative standard deviation (RSD) is less than or equal to 30% of QC samples and the sum of total strength values is greater than 70%, indicating the analysis system has good stability and that the data can be used for subsequent analysis. Pearson correlation analysis was performed on the strength values of QC samples, as shown in Fig. S1. The results suggest that the correlation coefficient was greater than 0.99 in both positive and negative models, indicating a significant correlation and allowing for further analysis of the data generated from MS. The quantity and identification of compounds after data pretreatment are shown in Table 1. There were 2,786 and 1,250 metabolic compounds with identification information in positive and negative ion mode, respectively. The principal component analysis (PCA) analysis in QC samples of positive and negative ion modes shows that the data collection process is reliable, shown in Fig. S2a and b. Meanwhile, the LB and IB groups are significantly distinguished from the control group in both positive and negative ion modes, respectively. These results indicate significant differences in metabolic profiles with LB and IB treatment when compared with control group. It is also indirectly proved that both living bacteria and inactivated bacteria have a major influence on the metabolism of microbiota in mice. However, the fecal metabolic profiles with LB and IB treatment are not clearly distinguished, which implies that they have similar metabolic profiles.

**TABLE 1:** The compound with identification information

Mode	Number of compounds^*a*^	Number of compounds with identification information^*b*^
Pos	5,639	2,786
Neg	2,390	1,250

^*a*^ The number of compounds represents the total count of ions that are collected in positive and negative ion modes by MS.

^*b*^ Compounds with identification information indicate that they can be identified at levels 1–4.

**Fig 4:**
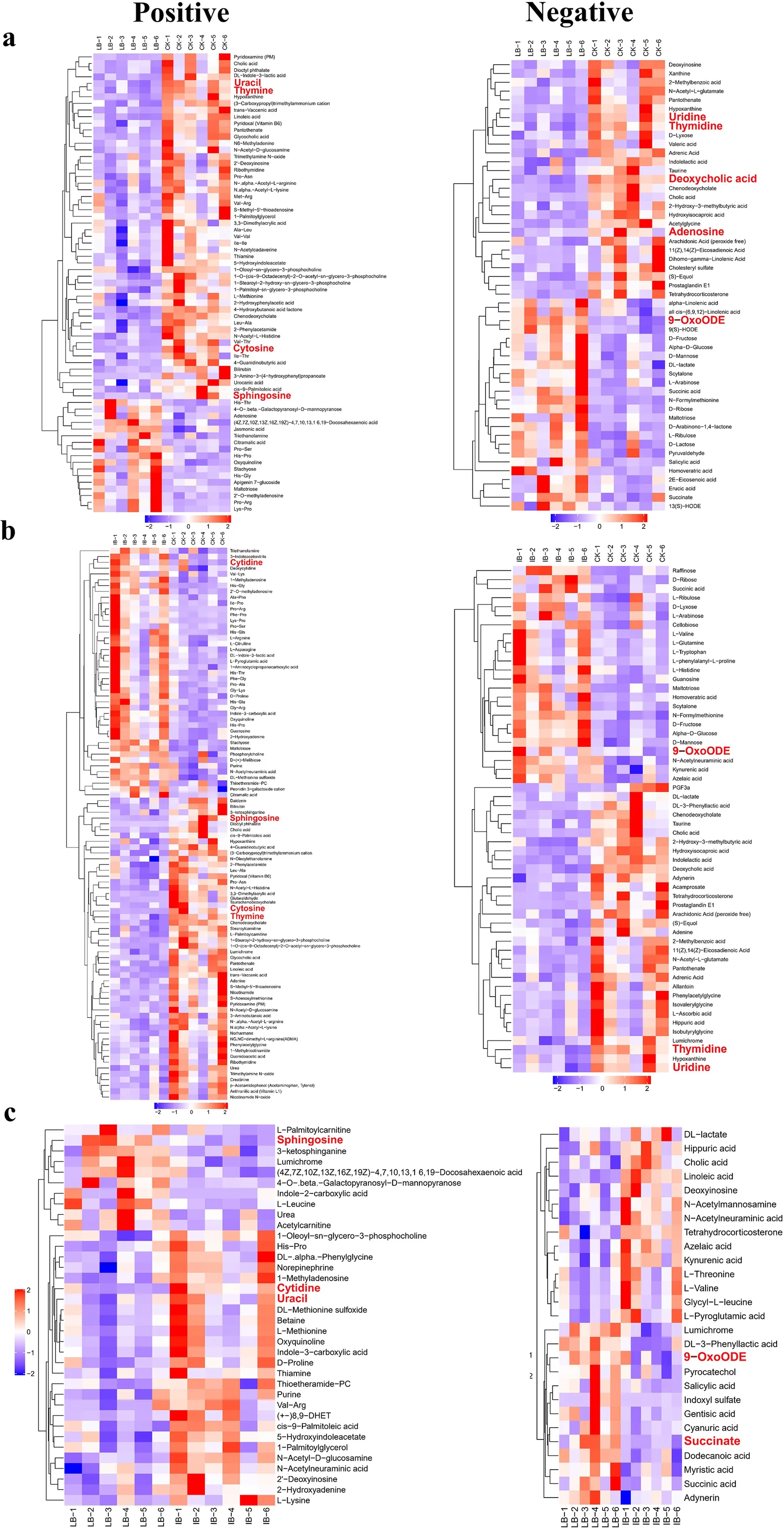
Analysis of metabolic profiles in fecal samples from mice with different treatments. Cluster analysis of differential metabolites for LB vs tumor control (CK) (a), IB vs control (CK) (b), and LB vs IB (c) in positive and negative ions mode, respectively. Each row represents a differential metabolite, each column shows a sample, and the color represents the amount of expression, blue to red corresponding to the amount of expression from low to high.

Furthermore, the metabolites with significant difference are obtained (Table S1 to S3). To further study metabolites with a highly significant difference, stricter screening conditions are employed for metabolites that show significant differences (VIP > 3, FC > 1, or FC < 0.83 as well as *P* < 0.01). The cluster analysis of differential metabolites for all samples is shown in Fig. 4.

Compared to control group, the up-regulated metabolites with significant difference in the LB groups are 9-oxooDE (*P* = 4.45 × 10^−5^, VIP = 10.94), 9(S)-HODE (*P* = 0.00096, VIP = 17.20), and succinic acid (*P* = 0.015, VIP = 3.455). However, linoleic acid (*P* = 1.04 × 10^−5^, VIP = 3.45), pantothenate (*P* = 0.00016, VIP = 3.237), cholic acid (*P* = 0.006, VIP = 3.361), chenodeoxycholate (*P* = 2.92 × 10^−5^, VIP = 11.946), deoxycholic acid (*P* = 9.408 × 10^−6^, VIP = 21.118), 4-guanidinobutyric acid (*P* = 0.0021, VIP = 2.665), and sphingosine (*P* = 0.01, VIP = 4.366) are the main down-regulated significant different metabolites in colon cancer models in mice administrated with living bacteria. Moreover, there are significant differences in metabolites, including urea (*P* = 0.0003, VIP = 5.681), creatinine (*P* = 0.0024, VIP = 11.401), pantothenate (*P* = 0.00019, VIP = 3.006), 1-Methylnicotinamide (*P* = 0.0085, VIP = 6.287), cholic acid (*P* = 0.0085, VIP = 10.833), chenodeoxycholate (*P* = 0.000027, VIP = 12.171), deoxycholic acid (*P* = 9.56 × 10^−6^, VIP = 21.723), thymine (*P* = 0.000046, VIP = 3.745), uracil (*P* = 0.00277, VIP = 5.098), cytosine (*P* = 0.0036, VIP = 5.985), linoleic acid (*P* = 5.703 × 10^−6^, VIP = 3.212), anthranilic acid (*P* = 0.0066, VIP = 8.311), and sphingosine (*P* = 0.0063, VIP = 4.96), that show lower expression level in IB group than tumor control group. In contrast, the significantly up-regulated different metabolites in the IB groups are maltotriose (*P* = 1.65 × 10^−6^, VIP = 3.133), D-(+)-Melibiose (*P* = 0.0022, VIP = 3.76), and Alpha-D-Glucose (*P* = 0.0022, VIP = 6.911) compared to the tumor control group. In comparison to IB group, succinate (*P* = 0.0056, VIP = 4.285) was up-regulated; while the metabolites, including *N*-acetyl-D-glucosamine (*P* = 0.0015, VIP = 3.050) and cytidine (*P* = 0.010, VIP = 1.714), were down-regulated.

Additionally, the results of Kyoto Encyclopedia of Genes and Genomes (KEGG) enrichment analysis indicate that significantly different metabolites associated with colon cancer progression are enriched in bile secretion, central carbon metabolism in cancer, phenylalanine metabolism and alanine, arginine and proline metabolism, as well as apoptosis process in control_LB groups, respectively (Fig. S3). In the control_IB groups, the significantly different metabolites are enriched in bile secretion, pyrimidine metabolism, apoptosis, arginine, and proline metabolism, respectively (Fig. S4). While the significantly different metabolites are enriched in pyrimidine metabolism in the LB_IB groups (Fig. S5).

### Coalition analysis of microbiome and metabolomics

**Fig 5:**
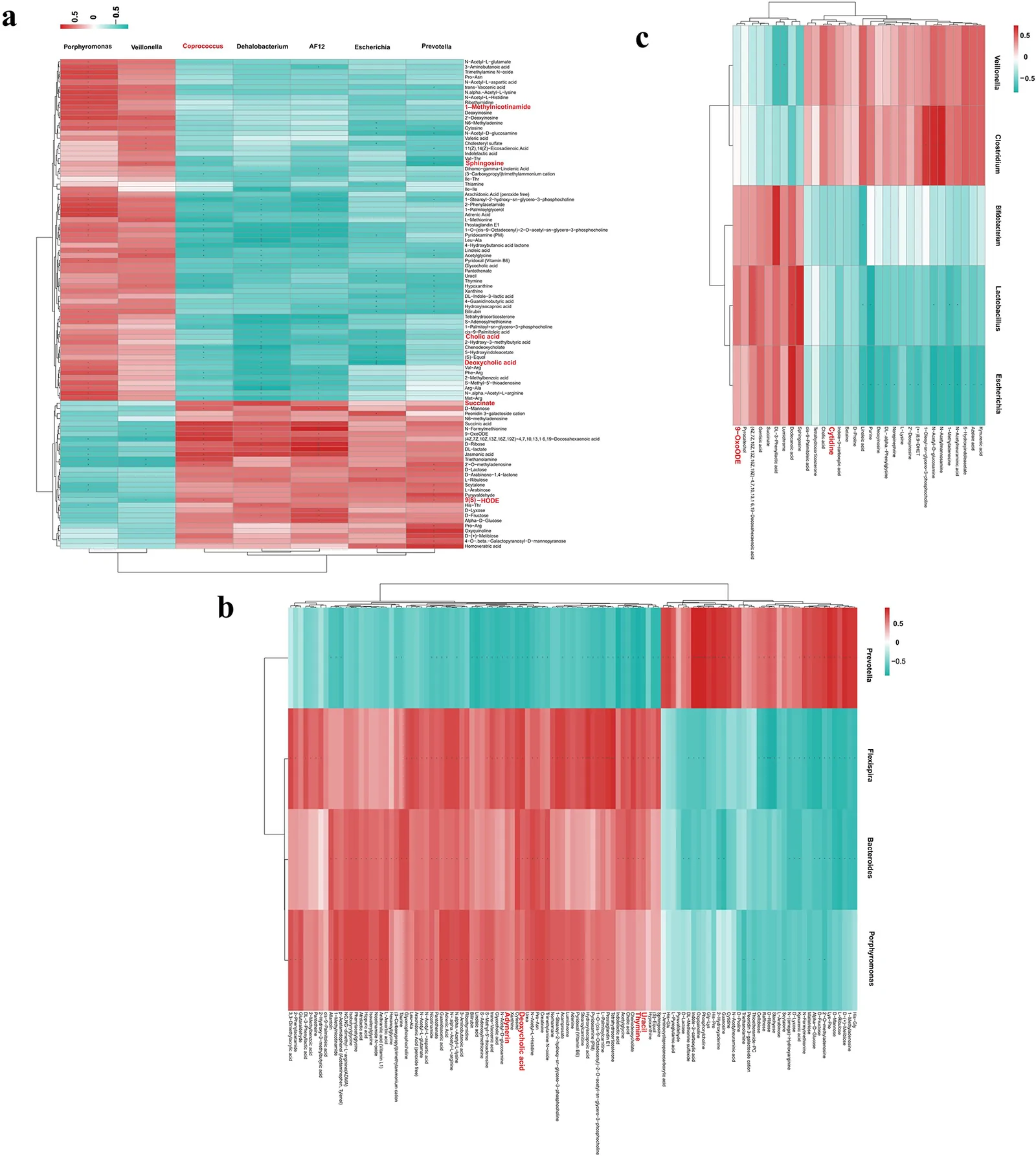
Correlation analysis of 16S microbial diversity and metabolomics with different treatments. The hierarchical cluster heat map of spearman correlation analysis on significant difference microbiota and metabolites in control_LB groups (a), control_IB groups (b), and LB_IB groups (c), respectively. Each row in the hierarchical cluster heat map represents a significant difference in metabolite, and each column represents a significant difference in microbiota at genus level. The correlation coefficient *R* is shown in color, with *R* > 0 indicating positive correlation as shown in red, and *R* < 0 representing a negative correlation as shown in blue. The deeper the color, the stronger the correlation.

In order to obtain a comprehensive understanding of interaction among microbiota, its metabolites, and progression of colon cancer under different treatments, a coalition analysis of the 16S microbial diversity and metabolomics are performed (Fig. 5).

In the control_LB groups, Spearman correlation analysis revealed significant positive correlations between sphingosine involved in apoptosis and *Veillonella*, as well as negative correlations with *Coprococcus* and *Prevotella*. Additionally, cholic acid and chenodeoxycholate were found to be negatively correlated with *Dehalobacterium*. Furthermore, *Porphyromonas* is positively correlated with deoxycholic acid, while *Coprococcus* and *Dehalobacterium* are positively correlated with succinate (Fig. 5a). Similarly, the coalition analysis of significant difference in microbiota and metabolites between the control_IB groups shows that uracil, thymine, and cytosine, as the key metabolites involved in pyrimidine metabolism signaling pathway, have the same expression pattern (Fig. 5b and c). These are positively correlated with *Prevotella* and are negatively correlated with *Bacteroides* and *Flexispira*, respectively. Cholic acid, chenodeoxycholate, and deoxycholic acid show similar expression patterns and are positively correlated with *Prevotella, Flexispira, Bacteroides,* except for deoxycholic acid.

### *Lactobacillus* inhibits the progression of colon cancer by down-regulating glycine, serine, and threonine metabolism to reduce sphingolipids

**Fig 6:**
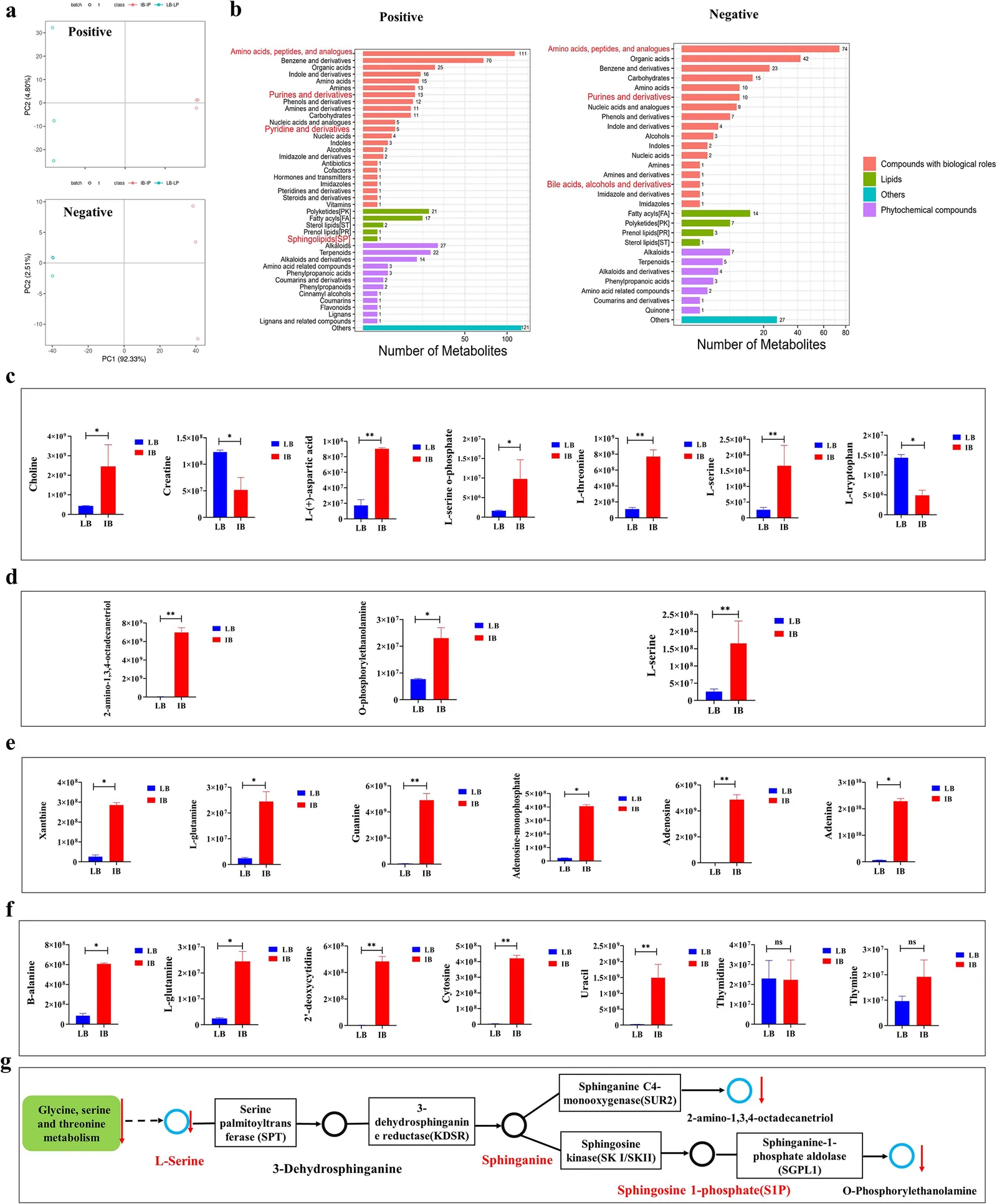
*Lactobacillus* inhibits the progression of colon cancer by down-regulating glycine, serine, and threonine metabolism. (a) Score plots of PCA for metabolites of LB culture supernatant and IB suspension solution. (b) Differential abundance analysis for metabolites of LB culture supernatant and IB suspension solution. (c) The metabolites enriched in LB culture supernatant and IB suspension solution, which are involved in glycine, serine, and threonine metabolism. (d) The metabolites enriched in LB culture supernatant and IB suspension solution, which are involved in sphingolipids [SP] metabolism. (e) The metabolites enriched in LB culture supernatant and IB suspension solution, which are involved in purine metabolism. (f) The metabolites enriched in LB culture supernatant and IB suspension solution, which are involved in pyrimidine metabolism. (g) Serine restriction down-regulated sphingolipid metabolism, the blue circle in figure represents the measured down-regulation metabolites.

To further identify metabolites with anticolon cancer properties produced by living bacteria (LB) and inactivated bacteria (IB), we performed nontargeted LC-MS/MS analysis on the culture supernatant of LB and the suspension solution of IB (Table S4; Fig. 6).

Score plots of PCA show that there are clearly distinguished metabolites between LB culture supernatant and IB suspension solution (Fig. 6a). Differential abundance analysis demonstrates that the critical products generated from living bacteria may contribute to the antitumor effects (Fig. 6b). Compared to IB group, the differential metabolites of LB are enriched in amino acids, peptides, and analogs; purines and derivatives; pyridine and derivatives; sphingolipids [SP] and bile acids; and alcohols and derivatives metabolism. This is further combined with the intestine metabolomics from colon cancer models in mice administrated with LB and IB. The metabolites involved in glycine, serine, and threonine metabolism, including choline, L-serine *o*-phosphate, creatine, L-threonine, L-(+)-aspartic acid, and L-serine, were significantly down-regulated in LB culture supernatant. In contrast, L-tryptophan was significantly up-regulated in LB culture supernatant compared to IB suspension solution (Fig. 6c). Additionally, the metabolites involved in sphingolipids [SP] metabolism, such as o-phosphorylethanolamine, 2-amino-1,3,4-octadecanetriol, and L-serine, are also significantly down-regulated in LB culture supernatant (Fig. 6d). The metabolites enriched in purine metabolism, including xanthine, L-glutamine, guanine, adenosine-monophosphate, adenosine, and adenine, are significantly down-regulated in LB culture supernatant in comparison to IB suspension solution (Fig. 6e). Similarly, pyrimidine metabolism in LB culture supernatant is significantly down-regulated compared to IB suspension solution. The relative abundance of metabolites included Β-alanine, L-glutamine, 2′-deoxycytidine, cytosine, and uracil notably decreased in LB culture supernatant with the exception of thymidine and thymine (Fig. 6f). Serine palmitoyltransferase (SPT) catalyzes the *de novo* biosynthesis of sphingolipids by using L-serine as a substrate. SPT converts L-serine into 3-dehydrosphinganine, which is then further converted into sphinganine/dihydrosphingosine by 3-dehydrosphinganine reductase (KDSR) (Fig. 6g). Ultimately, sphingosine kinase I/II (SK I/SK II) catalyzes the generation of sphingosine 1-phosphate (S1P) from sphinganine. Excitingly, sphinganine was significantly down-regulated in colon cancer models in mice treated with JY300-8 and JMR-01. Therefore, we anticipated that the down-regulation of key metabolites and metabolic pathway, including L-serine, alanine, adenine, uracil, and sphingolipids metabolism, as well as the enhancement of L-tryptophan metabolism were the main reasons for the antitumor effects with administration of probiotics.

## DISCUSSION

Recently, the relationship among altered gut microbiota, metabolites, and colon cancer has provided a potential strategy for preventing and treating colon cancer through modulation of the gut microbiota using probiotics (17, 18). Probiotics are the most commonly consumed food supplements due to their recognized safety and complete standardization (12). Thus, probiotics are considered as the most potentially safest agents for the prevention of cancer in the form of food supplements, especially colon cancer. Accumulating evidence shows that probiotics, such as *Lactobacillus*, could prevent animals that are susceptible to colon cancer against carcinoma effects *in vivo,* as shown in Table 2. The previous reported results demonstrate that both *Lactobacillus plantarum* and *Bacillus polyfermenticus* could exert limited antitumor efficiency, while they have no effect on the tumor formation rate (19, 20). The mixture of probiotic (*L. acidophilus, L. casei,* and *L. lactis biovar diacetylactis DRC-1*) reduced the tumor formation rate in DMH-induced colon cancer models in rat (40% of tumor formation rate in probiotic group vs 86.67% of that in control group) (21). Similarly, the tumor formation rate was significantly decreased in the *L. plantarum* YYC-3 treatment group (22.22% of tumor formation rate) compared to the control group (88.33% of tumor formation rate) (22). Although the highest protective effects are achieved in animal models treated with probiotic VSL#3 (0% of tumor formation rate), only 29% of animals developed carcinoma in control group (23). Furthermore, many probiotics only exert protective effects against colon cancer. However, they do not act on pre-existing colon cancer (24). Fortunately, our previous studies have verified that *L. casei* JY300-8 can inhibit the progression of pre-existing colon cancer (15). Similarly, the current results demonstrate that *L. casei* JY300-8 and *L. reuteri* JMR-01 not only significantly reduce the tumor formation rate (13.33% in LB vs 96.67% in control), but also remarkably suppress tumor growth and enhance the survival of CT26 cells-induced colon cancer models in mice. Therefore, oral administration of living JY300-8 and JMR-01 presents more potentially protective effects against the occurrence and progression of colon cancer in mice, which are considered as more promising probiotics for the prevention and therapy of colon cancer.

**TABLE 2:** Effects of probiotics on colon cancer models in animals

Strains	Colon cancer models	Antitumor efficiency	Reference
VSL#3	Colitis-associated colorectal cancer in rats	None of the probiotic-treated animals developed carcinoma (0 of tumor formation rate in VSL#3 vs 29% of that in control)	(23)
Heat-killed cells of *E. faecalis*	DSS-induced colitis in mice	*E. faecalis* may exert protective effects against CRC, but does not act on pre-existing CRC	(24)
*L. plantarum*	CT26 tumor-bearing mice	Although there is no effect on the tumor formation rate, the tumor volume reduces by 64.38% with oral administration of *L. plantarum*	(19)
*B. polyfermenticus*	Mouse xenograft model of human colon cancer	*B. polyfermenticus* conditioned medium (CM) reduces tumor growth and tumor proliferation	(20)
*L. acidophilus, L. casei, L. lactis biovar diacetylactis* DRC-1	DMH-induced colon cancer models in rats	Reduction in the tumor formation rate (40% of tumor formation rate in probiotic curd group vs 86.67% of that in control)	(21)
*L. plantarum* YYC-3	In the APC^Min/+^ mouse model of colon cancer	Reduction in the tumor formation rate (22.22% in YCC-3 group VS 88.33% in control)	(22)
JY300-8 and JMR-01	CT26 cells injection in mice	Reduction in the tumor formation rate (13.33% of tumor formation rate in LB group vs 96.67% of that in control)	In this study

Probiotics can regulate the structure and composition of gut microbiota, thereby modulating the occurrence and development of colon cancer (25, 26). The present research indicates that the composition and abundance of gut microbiota are associated with the progression of colon cancer through the administration of living or inactivated probiotics. *Flexispira, Bacterodes, Porphyromonas, Clostridium,* and *Escherichia* enriched in tumor control, especially *Clostridium and Escherichia,* are extensively existed in all of the tumor-bearing mice, which may play an important role in the progression of colon cancer models in mice. Similarly, recent research suggests that *Fusobacterium nucleatum, Escherichia coli,* and *Bacteroides fragilis* promote neoplastic processes in epithelial cells (27). We speculate that some microbes might be associated with colon cancer, including *Flexispira* and *Porphyromonas,* which possibly secrete the harmful metabolites, such as cholic acid, chenodeoxycholate, and deoxycholic acid. In addition, *Coprococcus, Veillonella, Lactobacillus, Bifidobacterium,* and *Dehalobacterium* in oral administration of living JY300-8 and JMR-01 are likely to reduce the occurrence and development of colon cancer. The previous evidence suggests that *Coprococcus* produced beneficial metabolites, such as short chain fatty acids, exerting the antitumor efficiency (28). Meanwhile, *Veillonella* could generate the acetate and propionate by the fermentation of lactate by *Lactobacillus* (29). The *prevotella* that is associated with gut inflammation (30) is enriched in administration of the inactivated JY300-8 and JMR-01. While the *prevotella* is reduced when orally administrated with living JY300-8 and JMR-01, which is consistent with previous reports that an overgrowth of *Prevotella* is correlated with a reduction of *Lactobacillus* (31). In summary, the altered microbiota is associated with colon cancer. *Flexispira*, *Bacterodes*, *Porphyromonas*, *Clostridium,* and *Escherichia* may play an important role in the progression of colon cancer. Conversely, *Coprococcus, Veillonella, Lactobacillus, Bifidobacterium,* and *Dehalobacterium* may exert the protective effects on susceptible mice for colon cancer.

Microbial metabolites in the gut play an important role in the progression of colon cancer (16). Recent evidence suggests that bile acids, especially deoxycholic acid (DCA), have been implicated in carcinogenesis of intestine due to their generation of ROS and reactive nitrogen species (RNS), both of which cause DNA damage (32, 33). In our study, bile acids including cholic acid (CA) and deoxycholic acid (DCA) were found to be extensively enriched in the intestine of mice in tumor control group. However, the levels of bile acids significantly decrease with the oral administration of living JY300-8 and JMR-01, or inactivated JY300-8 and JMR-01, which shows that the increased bile acids are positively correlated with the incidence and progression of colon cancer in mice. In addition, sphingosine is significant up-regulated in tumor control group, while it remarkably decreased with the intervention of living bacteria or inactivated bacteria. Sphingosine-1-phosphate (S1P), formed by the phosphorylation of sphingosine via the sphingosine kinase1 (SK1) and/or sphingosine kinase2 (SK2) *in vivo*, promotes tumorigenesis, transformation of normal cells to cancerous cells, neovascularization which provides cancer cells with nutrients and oxygen, tumor cell growth, and survival (34). The reported research demonstrates that higher levels of sphingosine can be used as selective pressure to increase SK1 expression, thereby converting sphingosine into S1P to promote the growth of tumor cell (35). Therefore, therapeutics strategies based on S1P pathway have been adopted to limit the effects of S1P signaling in cancer, including the reduction of the released S1P (36), inhibition of SK1 and/or SK2 (37) and targeting of specific S1P receptors (38). Importantly, in our study, the sphingosine in the intestine of tumor-bearing mice significantly decreased after oral administration of living or inactivated bacteria (JY300-8 and JMR-01), thus exerting antitumor efficiency. Therefore, the results imply that excellent probiotics can regulate sphingosine in the tumor microenvironment, which provides a potential therapeutic strategy by altering S1P signaling in cancer. Moreover, other microbial metabolites in oral administration of living bacteria (JY300-8 and JMR-01), such as succinic acid (28) and 9-OxoODE (39), exert anticarcinogenic activity. A similar report suggests that conjugated linoleic acids, such as 9-OxoODE, are produced from linoleic acid by the fermentation of *Lactobacillus* and *Bifidobacterium* in the intestinal lumen, thus presenting its beneficial effects (40). Additionally, pyrimidine metabolism, including thymine, cytosine, and uracil, is a critical pathway for DNA replication and RNA synthesis. Therefore, increased pyrimidine metabolism could guarantee uncontrolled growth of tumors (41). In our work, we demonstrate that the thymine, uracil, and cytosine, implicated in pyrimidine metabolism pathway, are depleted when administrated with the living and inactivated bacteria (JY300-8 and JMR-01) for colon cancer models in mice. Consistent with our results, changes in pyrimidine metabolism regulate proliferation of cancer cell (42). Therefore, the link between pyrimidine metabolism and tumorigenesis might provide novel targets for anticancer therapy through modulation of microbiota and its bioactive metabolites using the excellent probiotics.

The antitumor mechanisms influenced by microbiota and their metabolites with intervention of probiotic are always a hot topic. The metabolism of serine, glycine, and threonine plays a critical role in tumor growth, and limiting these processes is emerging as a potential therapeutic strategy for controlling tumorigenesis and progression of cancer (43 – 45). Living *L. casei* JY300-8 and *L. reuteri* JMR-01 restrict the level of L-serine through two metabolic pathways. One pathway is the down-regulation of synthesis of L-serine by reducing the choline, L-serine *o*-phosphate, creatine, L-threonine, and L-(+)-aspartic acid. Another is up-regulation of the degradation process of L-serine to produce more L-tryptophan. L-tryptophan is an important contributor in maintaining intestinal homeostasis and regulating innate immunity in the intestine (46). Meanwhile, L-tryptophan is commonly converted into its downstream metabolites by gut microbiota, thus presenting the positive functions by enriching the relative abundance of probiotics and depleting potential pathogens for colon cancer (47). These positive effects are similar to the compositional alterations observed in the gut microbiota of colon cancer models in mice following oral administration of living bacteria (JY300-8 and JMR-01). Particularly, serine restriction via JY300-8 and JMR-01 diminish sphingolipid metabolism, which down-regulates the key metabolic pathway of S1P signaling in cancer and inhibits colonic tumorigenesis and progression. Additionally, purine and pyrimidine metabolism, including adenine, cytosine, and uracil, is a critical pathway for DNA replication and RNA synthesis. Thereby, reduction of purine and pyrimidine metabolism could slow the growth of cancer. B-alanine, one of the key substrates of pyrimidine metabolism, decreases in LB culture supernatant, which is the main reason for the down-regulation of the pyrimidine metabolism. Nevertheless, alanine, serine, and glycine are widely existed in eggs, milk, meat, fish, shellfish, legumes, nuts, the whole grains, and vegetables. It is difficult in restricting the intake of these amino acids by limiting the human diet for cancer patient. Fortunately, the probiotics *L. casei* JY300-8 and *L. reuteri* JMR-01 can reduce the alanine levels and restrict both alanine and L-serine through the down-regulation of synthesis of L-serine and the up-regulation of the degradation process of L-serine to regulate the tumor microenvironment as well as suppressing the progression of colon cancer models in mice. Therefore, the limitation of alanine and L-serine processes with intervention of probiotic is an emerging therapy for colonic tumorigenesis and progression.

**Fig 7:**
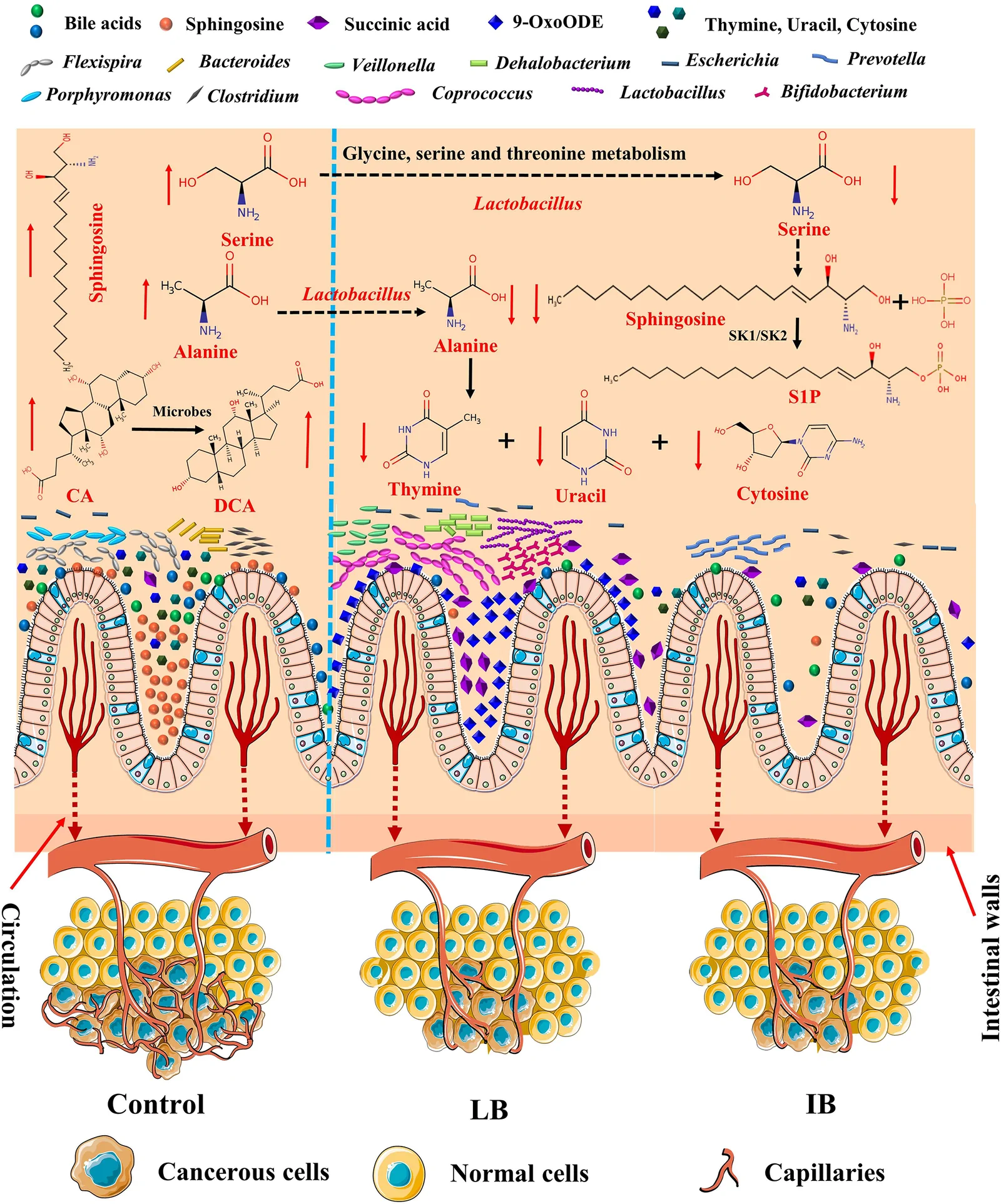
Graphical representation of major microbial and metabolomic alterations correlated with the tumorigenesis and the progression of colon cancer.

Based on the above findings, we have established a graphical representation of major microbial and metabolomic alterations that are correlated with the tumorigenesis and progression of colon cancer models in mice (Fig. 7). The present study on the correlation among the gut microbiome, colonic tumorigenesis, and progression reveals information about microbiota and its metabolite profiles in colon cancer. Our results suggest that microbiome and metabolome shift with different treatments. The characteristic microbiota of colon cancer includes *Flexispira, Bacterodes, Porphyromonas, Clostridium,* and *Escherichia*. Correspondingly, the characteristic metabolites related to colon cancer are obtained, including an increase in sphingosine, cholic acid (CA), deoxycholic acid (DCA), serine, and alanine, which can enter the small intestine capillaries and then reach the tumor tissue by the blood circulation, thereby promoting tumor growth. However, oral pre-supplementation with the excellent probiotic *Lactobacillus* mitigates the cancer-promoting effects by down-regulating cancer-associated intestinal microorganisms and metabolites in subcutaneous colon cancer models in mice. The beneficial microbiota, including *Coprococcus, Veillonella, Lactobacillus*, *Bifidobacterium,* and *Dehalobacterium*, increase when orally administrated with living JY300-8 and JMR-01. Meanwhile, *Lactobacillus* (JY300-8 and JMR-01) depleted serine by glycine, serine, and threonine metabolism pathway, thus reducing the relative abundance of sphingosine in intestine of colon cancer models in mice. Then, the reduction of sphingosine down-regulates the synthesis of S1P to modulate the S1P signal in cancer, thereby constraining the colonic tumorigenesis, reducing the transformation of the normal cells into cancerous cells and neovascularization. In addition, administration of *Lactobacillus* (JY300-8 and JMR-01) results in a decrease in the alanine, which leads to a reduction in the thymine, uracil, and cytosine. Thereby, the growth of cancer has been slowed down due to the reduction of key substrates in DNA replication and RNA synthesis by down-regulating purine and pyrimidine metabolism pathway. Additionally, reducing cancer-promoting metabolites, especially DCA, and producing antitumor compounds (9-OxoODE) regulate the tumor microenvironment, thereby inhibiting colonic tumorigenesis and progression.

### Conclusions

Our work demonstrated that *Lactobacillus* (JY300-8 and JMR-01) significantly constrain colonic tumorigenesis and progression. The tumor formation rate was reduced by 86.21% and 82.76%, and the tumor volumes declined by 65.2% and 61.18% when administrated with living or inactivated JY300-8 and JMR-01 in comparison to tumor control group, respectively. Meanwhile, the survival rates of tumor-bearing mice were 93.33% in both tumor control group and IB group, compared to a survival rate of 100% in the LB group at 30 days. Therefore, living *L.casei* JY300-8 and *L. reuteri* JMR-01 had more advantages in protecting against colonic tumorigenesis and progression than IB group. In addition, JY300-8 and JMR-01 reduced the relative abundance of L-serine through glycine, serine, and threonine metabolism to down-regulate sphingolipid metabolism and decrease sphingosine 1-phosphate signaling in mice, thereby exerting anticolon cancer effects. Similarly, depletion of L-serine via JY300-8 and JMR-01 could increase the L-tryptophan that regulated the intestinal homeostasis and increased the abundance of beneficial bacteria to produce the anticarcinogenic compounds. Moreover, JY300-8 and JMR-01 depleted B-alanine, one of the key substrates of pyrimidine metabolism, thus down-regulating the pyrimidine metabolism to constrain growth of tumor by decreasing cytosine and uracil. Therefore, modulation of gut microbiota and metabolites with intervention of probiotics could be a potential therapeutic strategy for preventing and treating colon cancer.

## MATERIALS AND METHODS

### Cells and probiotic preparation

The murine colon cancer cell line CT26 was purchased from Shanghai Cell Bank and cultured in RPMI1640 containing 10% of fetal bovine serum and 1% of penicillin/streptomycin at 37°C with a 5% CO_2_ of carbon dioxide incubator. The human colorectal adenocarcinoma cell lines HT29 and HCT116 were generously donated by the Biomedical Center of Institute of Modern Physics, Chinese Academy of Sciences. These colon cancer cells were cultured in DMEM containing 10% fetal bovine serum with a carbon dioxide incubator (5% CO_2_ and 95% air).

*Lactobacillus reuteri* JMR-01 (NCBI Accession No. MT362007) was isolated from the feces of the cured mice with breast cancer. *Lactobacillus casei* JY300-8 (15) is preserved in the Institute of Modern Physics, Chinese Academy of Sciences (IMP-CAS). JMR-01 or JY300-8 is statically cultured at 37°C for 24 h in MRS broth, and their fermentation solutions are subsequently centrifuged at 4,000 r/min for 10 min to acquire bacterial precipitates, respectively. Then, bacterial precipitates of JY300-8 and JMR-01 were resuspended in PBS at 1 × 10^9^ CFU/mL of concentration to obtain living JY300-8 and JMR-01, respectively. Subsequently, the living bacteria (LB) were prepared by the mixture of JY300-8 and JMR-01 at the ratio of 1:2 (vol/vol). In addition, LB was autoclaved at 105°C for 30 min to prepare an inactivated bacteria (IB) suspension solution.

### The analysis of antitumor efficacy of *Lactobacillus*

#### The effect of *Lactobacillus* on colon cancer cells *in vitro*

The effect of *Lactobacillus* on the proliferation of CT26, HT29, and HCT116 cells was studied by MTT assay (15), respectively. The logarithmic phase of colon cancer cells was incubated in 96-well plates with 100 µL of 1 × 10^6^ cells/mL per well. Then 100 µL of LB that diluted in DMEM medium (1 × 10^6^ CFU/mL) were added to 96-well plates filled with colon cancer cells for co-cultivating in 5% CO_2_ incubator at 37°C for 24, 48, and 72 h, respectively. Before 4 h of the end of co-cultivation, 5 µL of 5 mg/mL MTT was supplemented to each well and incubated for an additional 4 h. After removing the culture liquid in each well, 150 µL of dimethyl sulfoxide was added to fully dissolve the crystallites. The optical density was measured at 570 nm and the inhibitory rate was calculated as follows: inhibitory rate (%) = [ (1*a*) / *b*] × 100, where *a* is the absorbance value of experimental groups and *b* is absorbance value of colon cancer cells without co-cultivation of LB.

#### Experimental animals and the establishment of colon cancer models in mice

BALB/c mice (male, 6 wk) are purchased from Lanzhou Veterinary Research Institute, Chinese Academy of Agricultural Sciences. The mice were maintained on sterilized mice chow and water under constant temperature (20 ± 3°C) and 40–70% of humidity conditions with a 12/12 h light/dark cycle in a specific pathogen-free facility after approval by Institutional Animal Care and Use Committee at Biomedical Center, IMP-CAS. In accordance with the study schedule, the mice are euthanized at the end of the experiment. All animal experiments comply with the ARRIVE guidelines and are carried out in accordance with the National Institutes of Health guide for the care and use of Laboratory animals (NIH Publications No. 8023, revised 1978).

A subcutaneous colon cancer model is developed using CT26 cell in mice. CT26 cell are cultured in RPMI1640 medium containing 10% FBS at 37°C. When the cells reach exponential growth phase, 5 × 10^6^ of them are suspended in 100 µL cold PBS and subcutaneously injected into the right groin of mice. Then tumor formation rate, tumor volume, and survival rate are evaluated every other day with calipers. The tumor formation rate in treatment groups is determined by calculating the proportion of mice with a tumor volume equal to or greater than 100 mm^3^ among all mice, when the tumor models are successfully established in the control group. Tumor volume is 1/2 × *a* ^2^ *b*, where *a* and *b* represent the largest and shortest tumor diameters, respectively.

#### Experimental design and antitumor efficacy of *Lactobacillus*

Overall, the experimental scheme consists of two processes, including the prevention and treatment of subcutaneous colon cancer models in mice. Throughout the experiment, the BALB/c mice are randomly divided into three groups with 30 animals each group, including tumor control (CK) group, living bacteria (LB) group, and inactivated bacteria (IB) group. During the prevention stage, mice in control, LB and IB group, were subjected to semidiurnal gavage with 100 µL/head of PBS, 100 µL/head of LB, 100 µL/head of IB for 20 days, respectively. Subsequently, subcutaneous colon cancer models were induced by CT26 cells in mice. Meanwhile, each group of mice consistently underwent the above-mentioned prevention treatment. The tumor formation rate and the survival rate were evaluated when tumor models successfully established in control group (with a mean tumor volume of approximately 100 mm^3^). Then, during the treatment stage of colon cancer, tumor-bearing mice continued to be administered with PBS, LB, and IB until the end of the experiment. The tumor volume and survival rate are evaluated every other day. Fecal samples are collected to analyze the abundance of microbiota and its metabolome when a highly significant difference (*P* < 0.001) in the tumor volume is achieved between control group and treatment groups.

#### Sample collection and detection based on the animal models

To assess preventive effect of *Lactobacillus* on colon cancer models induced by CT26 cells in mice, the survival and tumor formation rates are measured in different groups after successful establishment of tumor models in the control group. To evaluate the therapeutic efficacy of *Lactobacillus* on colon cancer models in mice, the tumor volume and survival are monitored during treatment. Then, fecal sample in various groups are aseptically collected for analysis of the potentially altered microbiota and its metabolites by the intervention of LB or IB. Each group randomly chooses six fecal samples from different individuals to measure the microbiota and corresponding metabolites.

### Microbiota sequencing of fecal samples

Total genome DNA is extracted from fecal samples using TIANamp Bacteria DNA Kit (TIANGEN Biotechnology, China) according to the manufacturer protocol. The extracted DNA is then processed on an Illumina Miseq platform (48). The V3–V4 region of the 16S ribosomal RNA gene is amplified using sequence-specific primers (341F: CCTAYGGGRBGCASCAG, 806R: GGACTACNNGGGTATCTAAT). All PCR reactions are carried out in 30 µL reactions with 15 µL of Phusion High-Fidelity PCR Master Mix (New England Biolabs). The PCR products are mixed in equidensity ratios and purified using the AxyPrep DNA Gel Extraction Kit (AXYGEN). Subsequently, sequencing libraries are generated using NEB NextUltra DNA Library Prep Kit for Illumina (NEB, USA) according to manufacturer’s recommendations. The library quality is assessed using the Qubit@ 2.0 Fluorometer (Thermo Scientific) and Agilent Bioanalyzer 2100 system. Finally, the library is sequenced on an Illumina Miseq platform to obtain paired-end reads of 250/300 bp.

### Bioinformatic analysis of microbiota in fecal samples

Sequence analysis is performed by UPARSE software package using the UPARSE-OTU and UPARSE-OTUref algorithms. Sequences with a similarity of ≥97% are assigned to the same OTUs. Then, an OTU table and phylogenetic tree, as well as a UniFrac distance matrix, are obtained and weighted (49). The richness, inverse Simpson index, and Shannon index, are estimated using the phyloseq (version 1.30.0) and vegan (version 2.5–6) packages in R. Graphical representations of the relative abundance of bacterial diversity from phylum to species are visualized using Krona chart. Cluster analysis is preceded by PCA using the QIIME software package (50). LDA scores are calculated with LEfSe (51) using the factorial Kruskal–Wallis test (*P* < 0.05) to confirm the discriminative genera between groups, and a logarithmic LDA threshold score of 2.0 is set. Finally, the heatmap is drawn using heatmap package (version 1.10.12).

### Sample preparation and LC-MS analysis

#### Feces sample preparation and metabolite extraction

Metabolites in fecal samples are extracted according to previously reported methods (52). In short, 25 mg fecal samples are weighed and extracted by directly adding 800 µL of precooled extraction reagent [methanol:acetonitrile:water (2:2:1, vol/vol/vol)]. Then, 10 µL internal standards mix [L-Leucine-d3, L-PHENYLALANINE (13C9, 99%), L- Tryptophan-d5, and Progesterone-2,3,4–13C3] is added for quality. After homogenizing for 5 min using TissueLyser (JXFSTPRP, China) with the addition of two small steel balls, samples are processed by ultrasound at 4°C for 10 min and then incubated at −20°C for 1 h. After centrifugation for 15 min at 25,000 rpm at 4°C, the supernatant was subjected to vacuum drying. Subsequently, the metabolites are resuspended in 600 µL of 10% methanol and sonicated for 10 min at 4°C. Afterward, the supernatants are used for LC-MS analysis after centrifuging for 15 min at 25,000 rpm at 4°C. Additionally, 50 µL of the supernatant from each sample is mixed into a QC (quality control) sample to evaluate the repeatability and stability of the LC-MS analysis process.

#### *Lactobacillus* culture preparation and metabolite extraction

To investigate antitumor metabolites produced by living bacteria (LB) and inactivated bacteria (IB), the metabolism profiling of LB and IB are measured by liquid chromatography mass spectrometry (LC-MS/MS) and tandem mass spectrometry, respectively. JMR-01 or JY300-8 statically cultures at 37°C for 24 h in MRS broth, and its fermentation solution are centrifuged at 4°C 4,000 r/min for 10 min to obtain the bacteria culture supernatant. Then the culture supernatant of JMR-01 and JY300-8 is thoroughly mixed at volume of 2:1 (vol/vol) to prepare LB culture supernatant. Additionally, the living bacteria (LB) cell of JMR-01 and JY300-8 suspended in PBS buffer at the ratio of 2:1 (vol/vol) were autoclaved at 105°C for 30 min to prepare inactivated bacteria (IB) suspension solution. Subsequently, metabolites from LB culture supernatant and IB suspension solution were extracted, respectively. Following centrifugation, 20 µL of each sample (LB, IB) was added to a fresh glass vial for liquid chromatography mass spectrometry (LC-MS/MS) analysis according to the methods reported by Sugimura (47).

#### LC-MS analysis

The untargeted metabolites are analyzed using a Waters 2D UPLC (Waters, USA) tandem Q-Exactive high-resolution mass spectrometer (Thermo Fisher Scientific, USA) (53). Chromatographic separation was performed using a BEH C18 column (1.7 µm, 2.1 × 100 mm; Waters, USA) under the following conditions: for positive ion mode, the mobile phase consisted of 0.1% formic acid (solution A) and 100% methanol containing 0.1% formic acid (solution B); for negative ion mode, the mobile phase consisted of 10 mM ammonium formate (solution A) and 95% methanol containing 10 mM ammonium formate (solution B). The elution process was carried out using a B solution gradient, with the following conditions: 2% of B for 0–1 min, 2%–98% of B for 1–9 min, 98% of B for 9–12 min, and then returning to 2% of B from 12 to 12.1 min before ending at 2% of B from 12.1 to 15 min. The flow rate was maintained at a constant value of 0.35 mL/min and the column temperature remained at a steady state of 45°C throughout the experiment. Additionally, a 5 µL injection volume was utilized followed by primary and secondary mass spectrometry data collection using the Q-Exactive mass spectrometer (Thermo Fisher Scientific, USA). The mass-to-nuclear ratio for scanning ranged from 70 to 1,050 with a first-order resolution of 70,000. AGC was set at 3e6 and maximum injection time (IT) was limited to 100 ms. Fragmentation and secondary data acquisition were performed on Top3, based on parent ion intensity. The secondary resolution was set to 17,500 with an AGC of 1e5, while the maximum injection time (IT) was limited to 50 ms. Fragmentation energy (stepped normalized collisional energy [NCE]) was varied at levels of 20, 40, and 60 eV. For ESI ion source parameters, sheath gas flow rate and aux gas were maintained at rates of 40 and 10, respectively. In positive ion mode, spray voltage was set at a value of 3.80 kV, whereas in negative ion mode it was adjusted to a value of 3.20 kV. The temperature of capillary and aux gas heater was maintained at 320°C and 350°C, respectively (54, 55).

#### Data processing and statistical analysis of metabolomics data

MS-generated raw files for metabolites were processed by Compound Discoverer 3.1 (Thermo Fisher Scientific, USA). Then, exported compound from both positive and negative modes were filtered through several steps: (i) normalizing data by the Probabilistic Quotient Normalization (PNQ) and obtaining the relative peak areas (53); (ii) based on the QC sample information, calibrating the signal of the experiment sample by the quality control-based robust LOESS signal correction (QC-RLSC) (54); and (iii) excluding features with coefficient variance (CV) of QCs larger than 30% (55). Metabolome R software package and metaX are employed to perform data preprocessing, statistical analysis, metabolite classification, and functional annotation (56). We obtained metabolites from identification level 1 and level 4. Metabolites are identified by combining of house library (the BGI Library), Human Metabolome Database (HMDB), KEGG, and Lipid Maps databases (57). Subsequently, PCA is performed to analyze the similarities and differences within groups as well as outliers (58). Then, significant differential metabolites are screened by a variable that is important for calculating the projection (VIP) using partial least squares discriminant analysis (PLS-DA) (59) and combined with fold change (FC) of a single variable analysis and *P* value from Student’s *t*-test (60).

### Coalition analysis of 16S rDNA amplicon sequencing and untargeted metabolomics

Spearman statistical method is employed to analyze the correlation coefficient between the significant difference in microflora and significant difference in metabolites. Then, R and Cytoscape software are used to perform the analysis of the matrix heat map, hierarchical clustering, and correlation network to investigate the interaction relationship between microbiota and metabolites.

### Statistical analysis

Statistical analysis is performed using SPSS 22.0 software (SPSS Inc., Chicago, IL, USA), and the figure is plotted using Origin 2019 (OriginLab Corp., Northampton, MA, USA) and GraphPad Prism 8.0.1 software (GraphPad Inc., CA, USA). The significant differences between the two groups were analyzed using the *t*-test of independent samples by one-way ANOVA with the Tukey–Kramer comparison test. Statistical significance levels were indicated as ns, not significant, *P < 0.05, **P < 0.01, ***P < 0.001.

## Data Availability

The sequencing data of 16S rDNA in this study have been submitted in the NCBI database, and we have obtained the BioProject accession number: PRJNA807432.

## SUPPLEMENTAL MATERIAL

Additional file : Tables S1-S3 spectrum.00189-23-s0001.docx The metabolites with significant difference in various treatments.

Additional file : Fig. S1 spectrum.00189-23-s0002.jpg Correlation map of QC samples.

Additional file : Fig. S2 spectrum.00189-23-s0003.jpg PCA analysis of fecal metabolomic samples with various treatments in positive and negative ion model.

Additional file : Fig. S3 spectrum.00189-23-s0004.pdf KEGG enrichment analysis in control_LB groups.

Additional file : Fig. S4 spectrum.00189-23-s0005.pdf KEGG enrichment analysis in control_IB groups.

Additional file : Fig. S5 spectrum.00189-23-s0006.pdf KEGG enrichment analysis in LB_IB groups.

Supplemental legends spectrum.00189-23-s0007.docx Legends for supplemental figures and tables.

Additional file : Table S4 spectrum.00189-23-s0008.docx Differential changed metabolites between LB culture supernatant and IB suspension solution.

ASM does not own the copyrights to Supplemental Material that may be linked to, or accessed through, an article. The authors have granted ASM a non-exclusive, world-wide license to publish the Supplemental Material files. Please contact the corresponding author directly for reuse.
