# Supplementary material for: The efficacy of prevention for colon cancer based on the microbiota therapy and the antitumor mechanisms with intervention of dietary Lactobacillus
Source: Microbiol Spectr. 2023 Sep 1;11(5):e00189-23. doi: 10.1128/spectrum.00189-23 (PMC10581183; doi:10.1128/spectrum.00189-23)
Supplement: Additional file : Tables S1-S3 — The metabolites with significant difference in various treatments. [file spectrum.00189-23-s0001.docx]

**Table S1 Significant differences metabolites between LB and tumor control groups in positive and negative ions mode**

| **Description** | **VIP** | **Fold change** | **p-value** |
| --- | --- | --- | --- |
| Deoxycholic acid | 21.118 | 0.386 | 0.000009 |
| 9(S)-HODE | 17.207 | 2.121 | 0.0010 |
| Hypoxanthine | 15.338 | 0.346 | 0.00032 |
| Chenodeoxycholate | 11.946 | 0.371 | 0.00003 |
| Cholic acid | 11.647 | 0.219 | 0.0068 |
| 9-OxoODE | 10.943 | 2.539 | 0.000044 |
| (3-Carboxypropyl) trimethylammonium cation | 9.308 | 0.359 | 0.0016 |
| Hydroxyisocaproic acid | 7.557 | 0.326 | 0.0022 |
| Uracil | 6.578 | 0.467 | 0.0004 |
| Cytosine | 6.365 | 0.098 | 0.0043 |
| (S)-Equol | 6.281 | 0.512 | 0.0025 |
| Trimethylamine N-oxide | 6.071 | 0.393 | 0.0175 |
| S-Methyl-5'-thioadenosine | 6.025 | 0.383 | 0.0399 |
| Thiamine | 6.005 | 0.595 | 0.0455 |
| Hypoxanthine | 5.762 | 0.284 | 0.0018 |
| Pro-Arg | 5.566 | 3.440 | 0.0466 |
| 1-Methylnicotinamide | 5.429 | 0.428 | 0.0353 |
| Succinic acid | 5.374 | 2.954 | 0.0154 |
| Scytalone | 5.373 | 2.799 | 0.0170 |
| Alpha-D-Glucose | 5.236 | 2.086 | 0.0317 |
| 1-Palmitoyl-sn-glycero-3-phosphocholine | 5.159 | 0.702 | 0.0461 |
| Arachidonic Acid (peroxide free) | 4.827 | 0.557 | 0.0333 |
| Pantothenate | 4.582 | 0.267 | 0.0015 |
| Sphingosine | 4.366 | 0.381 | 0.0107 |
| Adrenic Acid | 4.120 | 0.360 | 0.0002 |
| Chenodeoxycholate | 3.996 | 0.437 | 0.0001 |
| Arg-Ala | 3.908 | 0.514 | 0.0457 |
| Homoveratric acid | 3.576 | 2.918 | 0.0435 |
| Ile-Ile | 3.485 | 0.570 | 0.0399 |
| Linoleic acid | 3.455 | 0.178 | 0.00001 |
| Thymine | 3.420 | 0.402 | 0.0004 |
| Cholic acid | 3.361 | 0.214 | 0.0060 |
| D- (+)-Melibiose | 3.242 | 2.720 | 0.0449 |
| Pantothenate | 3.237 | 0.311 | 0.00016 |
| Tetrahydrocorticosterone | 3.205 | 0.274 | 0.0031 |
| 1-Stearoyl-2-hydroxy-sn-glycero-3-phosphocholine | 3.081 | 0.213 | 0.0061 |
| N.alpha.-Acetyl-L-lysine | 3.042 | 0.382 | 0.0011 |
| N-alpha-Acetyl-L-arginine | 2.949 | 0.556 | 0.0307 |
| trans-Vaccenic acid | 2.901 | 0.208 | 0.0023 |
| Deoxyinosine | 2.862 | 0.444 | 0.0045 |
| N-Acetyl-L-Histidine | 2.861 | 0.382 | 0.0026 |
| cis-9-Palmitoleic acid | 2.832 | 0.662 | 0.0057 |
| Xanthine | 2.796 | 0.444 | 0.0066 |
| N-Acetyl-L-glutamate | 2.696 | 0.315 | 0.0070 |
| 4-Guanidinobutyric acid | 2.666 | 0.320 | 0.0022 |
| L-Arabinose | 2.574 | 2.066 | 0.0152 |
| Leu-Ala | 2.487 | 0.436 | 0.0029 |
| Val-Arg | 2.363 | 0.471 | 0.0359 |
| (4Z,7Z,10Z,13Z,16Z,19Z)-4,7,10,13,1 6,19-Docosahexaenoic acid | 2.329 | 3.033 | 0.0019 |
| Ribothymidine | 2.152 | 0.340 | 0.0241 |
| Ile-Thr | 2.105 | 0.517 | 0.0300 |
| Cholesteryl sulfate | 2.101 | 0.405 | 0.0258 |
| L-Methionine | 2.090 | 0.528 | 0.0129 |
| N6-Methyladenine | 2.029 | 0.362 | 0.0342 |
| 2-Methylbenzoic acid | 1.976 | 0.478 | 0.0235 |
| Pro-Asn | 1.956 | 0.516 | 0.0146 |
| 2-Hydroxy-3-methylbutyric acid | 1.948 | 0.535 | 0.0057 |
| N-Acetyl-D-glucosamine | 1.908 | 0.572 | 0.0216 |
| Triethanolamine | 1.881 | 1.258 | 0.0288 |
| Uracil | 1.867 | 0.419 | 0.00011 |
| Succinate | 1.853 | 1.907 | 0.0320 |
| Pyruvaldehyde | 1.834 | 2.536 | 0.0018 |
| Pyridoxal (Vitamin B6) | 1.820 | 0.570 | 0.0014 |
| Bilirubin | 1.817 | 0.276 | 0.0183 |
| Pyridoxamine (PM) | 1.802 | 0.268 | 0.0058 |
| Prostaglandin E1 | 1.789 | 0.242 | 0.0047 |
| Peonidin 3-galactoside cation | 1.767 | 1.493 | 0.0472 |
| Glycocholic acid | 1.721 | 0.238 | 0.00078 |
| DL-Indole-3-lactic acid | 1.721 | 0.445 | 0.0017 |
| D-Lactose | 1.714 | 4.737 | 0.0013 |
| 2'-O-methyladenosine | 1.675 | 2.406 | 0.0300 |
| N-Formylmethionine | 1.643 | 2.949 | 0.0023 |
| Val-Thr | 1.622 | 0.362 | 0.0148 |
| 3-Aminobutanoic acid | 1.622 | 0.558 | 0.0200 |
| His-Thr | 1.609 | 1.639 | 0.0402 |
| DL-lactate | 1.591 | 1.368 | 0.0061 |
| 4-O-beta-Galactopyranosyl-D-mannopyranose | 1.566 | 2.937 | 0.0475 |
| L-Ribulose | 1.515 | 3.370 | 0.0015 |
| Dihomo-gamma-Linolenic Acid | 1.423 | 0.260 | 0.0494 |
| D-Mannose | 1.421 | 2.626 | 0.0286 |
| D-Ribose | 1.406 | 2.094 | 0.0043 |
| 11(Z),14(Z)-Eicosadienoic Acid | 1.398 | 0.212 | 0.0251 |
| 2-Phenylacetamide | 1.393 | 0.532 | 0.0014 |
| 1-O-(cis-9-Octadecenyl)-2-O-acetyl-sn-glycero-3-phosphocholine | 1.345 | 0.520 | 0.0048 |
| 2'-Deoxyinosine | 1.336 | 0.561 | 0.0053 |
| Thymine | 1.309 | 0.582 | 0.00005 |
| 1-Palmitoylglycerol | 1.300 | 0.401 | 0.0263 |
| Met-Arg | 1.296 | 0.428 | 0.0334 |
| 5-Hydroxyindoleacetate | 1.218 | 0.631 | 0.0106 |
| D-Lyxose | 1.207 | 2.204 | 0.0193 |
| S-Adenosylmethionine | 1.178 | 0.416 | 0.0263 |
| N-Acetyl-L-aspartic acid | 1.146 | 0.486 | 0.0144 |
| Xanthine | 1.140 | 0.307 | 0.00092 |
| Oxyquinoline | 1.134 | 1.756 | 0.0213 |
| Jasmonic acid | 1.127 | 2.125 | 0.0017 |
| Phe-Arg | 1.119 | 0.520 | 0.0185 |
| D-Fructose | 1.116 | 2.266 | 0.0172 |
| N6-methyladenosine | 1.103 | 1.409 | 0.0187 |
| Valeric acid | 1.098 | 0.485 | 0.0326 |
| Acetylglycine | 1.098 | 0.229 | 0.0011 |
| D-Arabinono-1,4-lactone | 1.094 | 4.945 | 0.0031 |
| 4-Hydroxybutanoic acid lactone | 1.076 | 0.431 | 0.000003 |
| Indolelactic acid | 1.060 | 0.550 | 0.0412 |

**Table S2 Significant differences metabolites between IB and tumor control groups in positive and negative ions mode**

| **Description** | **VIP** | **Fold change** | **p-value** |
| --- | --- | --- | --- |
| Deoxycholic acid | 21.723 | 0.319 | 0.00001 |
| Chenodeoxycholate | 12.171 | 0.306 | 0.00003 |
| Creatinine | 11.402 | 0.472 | 0.0025 |
| Cholic acid | 10.834 | 0.229 | 0.0085 |
| Hypoxanthine | 8.774 | 0.530 | 0.0284 |
| 3-Carboxypropyl) trimethylammonium cation | 8.702 | 0.318 | 0.0008 |
| Anthranilic acid (Vitamin L1) | 8.312 | 0.455 | 0.0066 |
| Hydroxyisocaproic acid | 8.200 | 0.209 | 0.0008 |
| Trimethylamine N-oxide | 7.338 | 0.241 | 0.0018 |
| S-Methyl-5'-thioadenosine | 7.197 | 0.180 | 0.0058 |
| Alpha-D-Glucose | 6.912 | 2.800 | 0.0023 |
| Hippuric acid | 6.750 | 0.332 | 0.0181 |
| 1-Methylnicotinamide | 6.288 | 0.326 | 0.0085 |
| Scytalone | 6.242 | 3.419 | 0.0083 |
| (S)-Equol | 6.056 | 0.487 | 0.0023 |
| Cytosine | 5.985 | 0.076 | 0.0037 |
| Urea | 5.681 | 0.261 | 0.0003 |
| Arachidonic Acid (peroxide free) | 5.635 | 0.524 | 0.0147 |
| Uracil | 5.099 | 0.494 | 0.0028 |
| Lumichrome | 4.995 | 0.447 | 0.0004 |
| Sphingosine | 4.960 | 0.262 | 0.0037 |
| Phenylacetylglycine | 4.839 | 0.194 | 0.0281 |
| DL-3-Phenyllactic acid | 4.536 | 0.321 | 0.0203 |
| Hypoxanthine | 4.163 | 0.387 | 0.0129 |
| 3,3-Dimethylacrylic acid | 4.045 | 0.461 | 0.0221 |
| L-Pyroglutamic acid | 3.915 | 2.108 | 0.0499 |
| N-alpha-Acetyl-L-arginine | 3.855 | 0.469 | 0.0022 |
| Lys-Pro | 3.791 | 4.596 | 0.0403 |
| Pantothenate | 3.782 | 0.340 | 0.0029 |
| NG, NG-dimethyl-L-arginine (ADMA) | 3.770 | 0.424 | 0.0195 |
| D- (+)-Melibiose | 3.762 | 3.102 | 0.0022 |
| Thymine | 3.745 | 0.320 | 0.00005 |
| Chenodeoxycholate | 3.736 | 0.424 | 0.0001 |
| 2-Hydroxyadenine | 3.674 | 2.230 | 0.0241 |
| Adrenic Acid | 3.539 | 0.453 | 0.0006 |
| Nicotinamide N-oxide | 3.460 | 0.390 | 0.0091 |
| Allantoin | 3.339 | 0.421 | 0.0157 |
| Linoleic acid | 3.213 | 0.210 | 0.00001 |
| Maltotriose | 3.134 | 7.028 | 0.000002 |
| N.alpha.-Acetyl-L-lysine | 3.100 | 0.370 | 0.0002 |
| trans-Vaccenic acid | 3.100 | 0.124 | 0.0010 |
| Thioetheramide-PC | 3.049 | 1.614 | 0.0105 |
| N-Acetyl-L-Histidine | 3.028 | 0.287 | 0.0009 |
| Pantothenate | 3.006 | 0.323 | 0.0002 |
| Nicotinamide | 2.994 | 0.146 | 0.0131 |
| 1-Stearoyl-2-hydroxy-sn-glycero-3-phosphocholine | 2.976 | 0.187 | 0.0054 |
| Isovalerylglycine | 2.891 | 0.368 | 0.0190 |
| 1-Methyladenosine | 2.817 | 2.309 | 0.0034 |
| 1-Aminocyclopropanecarboxylic acid | 2.801 | 2.395 | 0.0431 |
| Guanosine | 2.724 | 2.364 | 0.0298 |
| Maltotriose | 2.666 | 11.829 | 0.0008 |
| Guanosine | 2.662 | 2.214 | 0.0221 |
| Tetrahydrocorticosterone | 2.562 | 0.377 | 0.0065 |
| N-Acetyl-L-glutamate | 2.464 | 0.315 | 0.0050 |
| Pro-Asn | 2.462 | 0.381 | 0.0011 |
| L-Arabinose | 2.425 | 2.025 | 0.0334 |
| Ribothymidine | 2.325 | 0.291 | 0.0153 |
| Pantetheine | 2.275 | 0.209 | 0.0488 |
| Norharmane | 2.200 | 0.685 | 0.0130 |
| Leu-Ala | 2.143 | 0.553 | 0.0104 |
| Acamprosate | 2.143 | 0.460 | 0.0116 |
| Guanidoacetic acid | 2.103 | 0.334 | 0.0079 |
| Phenylacetylglycine | 2.098 | 0.281 | 0.0319 |
| Isobutyrylglycine | 2.064 | 0.369 | 0.0179 |
| His-Pro | 2.053 | 2.407 | 0.0197 |
| 2-Hydroxy-3-methylbutyric acid | 2.035 | 0.494 | 0.0162 |
| Hexanoylglycine | 2.034 | 0.526 | 0.0307 |
| L-Palmitoylcarnitine | 2.018 | 0.424 | 0.0010 |
| Adenine | 1.991 | 0.404 | 0.0083 |
| 2-Methylbenzoic acid | 1.899 | 0.482 | 0.0231 |
| 3-Aminobutanoic acid | 1.887 | 0.497 | 0.0028 |
| L-Ascorbic acid | 1.862 | 0.394 | 0.0191 |
| Peonidin 3-galactoside cation | 1.851 | 1.582 | 0.0302 |
| N-Acetylneuraminic acid | 1.834 | 1.870 | 0.0280 |
| Taurine | 1.831 | 0.572 | 0.0276 |
| 2'-O-methyladenosine | 1.816 | 2.670 | 0.0062 |
| His-Gly | 1.789 | 2.433 | 0.0059 |
| Bilirubin | 1.785 | 0.248 | 0.0172 |
| Lumichrome | 1.774 | 0.578 | 0.0136 |
| Triethanolamine | 1.771 | 1.189 | 0.0164 |
| D-Mannose | 1.765 | 3.310 | 0.0006 |
| cis-9-Palmitoleic acid | 1.709 | 0.752 | 0.0394 |
| Pyridoxamine (PM) | 1.683 | 0.296 | 0.0074 |
| N-Acetylneuraminic acid | 1.606 | 1.988 | 0.0054 |
| Indolelactic acid | 1.601 | 0.284 | 0.00003 |
| Oxyquinoline | 1.598 | 2.846 | 0.0055 |
| Prostaglandin E1 | 1.588 | 0.287 | 0.0064 |
| Phosphorylcholine | 1.562 | 1.480 | 0.0015 |
| DL-Methionine sulfoxide | 1.534 | 1.634 | 0.0148 |
| Pyruvaldehyde | 1.526 | 2.126 | 0.0393 |
| Cytidine | 1.484 | 3.370 | 0.0263 |
| Gly-Lys | 1.481 | 1.993 | 0.0303 |
| Kynurenic acid | 1.479 | 1.288 | 0.0430 |
| Pyridoxal (Vitamin B6) | 1.466 | 0.651 | 0.0020 |
| His-Glu | 1.458 | 2.081 | 0.0157 |
| D-Ribose | 1.442 | 2.203 | 0.0026 |
| D-Proline | 1.436 | 1.574 | 0.0379 |
| D-Fructose | 1.425 | 2.984 | 0.0010 |
| Indole-3-carboxylic acid | 1.399 | 2.887 | 0.0012 |
| Stearoylcarnitine | 1.356 | 0.491 | 0.0025 |
| Glycocholic acid | 1.353 | 0.363 | 0.0028 |
| N-(omega)-Hydroxyarginine | 1.347 | 2.099 | 0.0276 |
| Atrolactic acid | 1.346 | 0.429 | 0.0317 |
| N-Acetyl-D-glucosamine | 1.343 | 0.304 | 0.0159 |
| D-Lyxose | 1.339 | 2.546 | 0.0145 |
| S-Adenosylmethionine | 1.337 | 0.236 | 0.0029 |
| Thymine | 1.337 | 0.517 | 0.0001 |
| Adynerin | 1.325 | 0.419 | 0.0063 |
| N-Formylmethionine | 1.323 | 2.072 | 0.0001 |
| Stachyose | 1.322 | 3.820 | 0.0001 |
| p-Acetamidophenol (Acetaminophen, Tylenol) | 1.314 | 0.272 | 0.0053 |
| 2-Phenylacetamide | 1.299 | 0.590 | 0.0092 |
| Raffinose | 1.275 | 2.875 | 0.0104 |
| D-Lactose | 1.275 | 3.299 | 0.0111 |
| L-Ribulose | 1.274 | 2.604 | 0.0009 |
| Glutaraldehyde | 1.227 | 0.470 | 0.0187 |
| N-Acetyl-L-aspartic acid | 1.216 | 0.398 | 0.0014 |
| Cellobiose | 1.114 | 2.207 | 0.0267 |
| 1-O-(cis-9-Octadecenyl)-2-O-acetyl-sn-glycero-3-phosphocholine | 1.110 | 0.562 | 0.0043 |
| Glycerophosphocholine | 1.080 | 0.633 | 0.0162 |
| Acetylglycine | 1.020 | 0.230 | 0.0011 |
| S-Methyl-5'-thioadenosine | 1.009 | 0.281 | 0.0006 |
| Xanthine | 1.001 | 0.364 | 0.0033 |

**Table S3 Significant differences metabolites between LB and IB groups in positive and negative ions mode**

| **Description** | **VIP** | **Fold change** | **p-val ue** |
| --- | --- | --- | --- |
| D-Proline | 8.481 | 0.635 | 0.024 |
| 9-OxoODE | 8.387 | 1.461 | 0.045 |
| DL-3-Phenyllactic acid | 6.006 | 2.068 | 0.004 |
| 1-Oleoyl-sn-glycero-3-phosphocholine | 5.801 | 0.687 | 0.025 |
| Lumichrome | 5.319 | 1.759 | 0.012 |
| Betaine | 4.796 | 0.634 | 0.045 |
| Lumichrome | 4.755 | 1.669 | 0.008 |
| Succinate | 4.285 | 1.930 | 0.006 |
| Cholic acid | 3.878 | 0.760 | 0.013 |
| Deoxyinosine | 3.517 | 0.672 | 0.044 |
| N-Acetyl-D-glucosamine | 3.050 | 0.641 | 0.001 |
| Sphingosine | 2.914 | 1.455 | 0.011 |
| Norepinephrine | 2.831 | 0.784 | 0.029 |
| 1-Methyladenosine | 2.614 | 0.576 | 0.019 |
| Gentisic acid | 2.424 | 1.618 | 0.028 |
| (4Z,7Z,10Z,13Z,16Z,19Z)-4,7,10,13,1 6,19-Docosahexaenoic acid | 2.132 | 1.998 | 0.019 |
| Azelaic acid | 2.093 | 0.685 | 0.014 |
| N-Acetylneuraminic acid | 2.065 | 0.615 | 0.006 |
| (+-)8,9-DHET | 1.992 | 0.423 | 0.045 |
| N-Acetylmannosamine | 1.968 | 0.688 | 0.012 |
| cis-9-Palmitoleic acid | 1.862 | 0.591 | 0.024 |
| Purine | 1.811 | 0.774 | 0.031 |
| Tetrahydrocorticosterone | 1.766 | 0.727 | 0.029 |
| Cytidine | 1.714 | 0.268 | 0.010 |
| DL-alpha-Phenylglycine | 1.560 | 0.777 | 0.036 |
| 2'-Deoxyinosine | 1.552 | 0.688 | 0.016 |
| Kynurenic acid | 1.409 | 0.793 | 0.041 |
| 5-Hydroxyindoleacetate | 1.404 | 0.795 | 0.012 |
| L-Lysine | 1.343 | 0.562 | 0.038 |
| Indole-3-carboxylic acid | 1.203 | 0.600 | 0.016 |
| N-Acetylneuraminic acid | 1.199 | 0.708 | 0.033 |
| Linoleic acid | 1.096 | 0.372 | 0.0001 |
| Dodecanoic acid | 1.051 | 1.295 | 0.004 |
| Pyrocatechol | 1.022 | 1.363 | 0.046 |
