# Supplementary figures and images for: The efficacy of prevention for colon cancer based on the microbiota therapy and the antitumor mechanisms with intervention of dietary Lactobacillus
Source: Microbiol Spectr. 2023 Sep 1;11(5):e00189-23. doi: 10.1128/spectrum.00189-23 (PMC10581183; doi:10.1128/spectrum.00189-23)

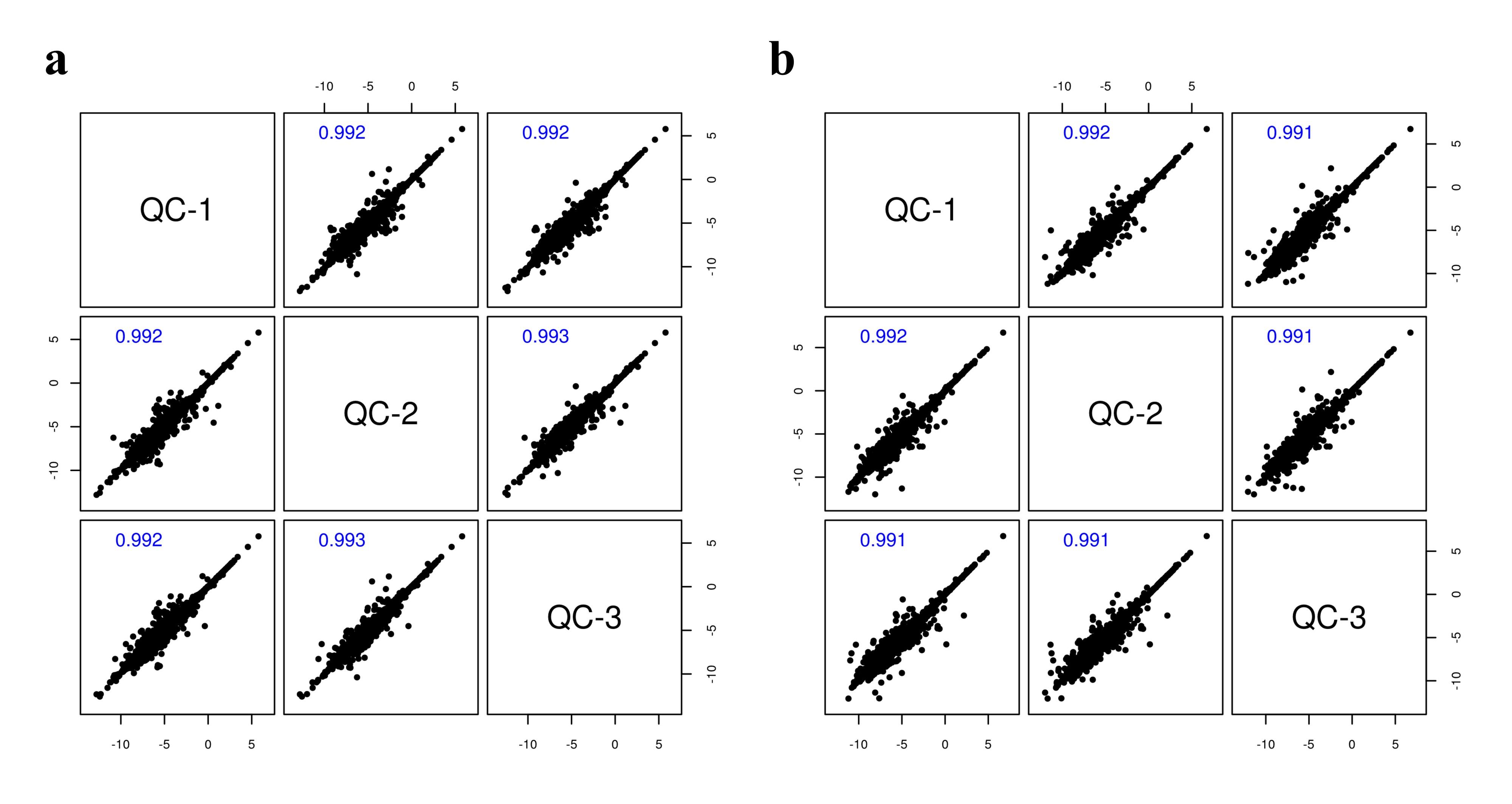

Supplement: Additional file : Fig. S1 — Correlation map of QC samples. [file spectrum.00189-23-s0002.jpg]

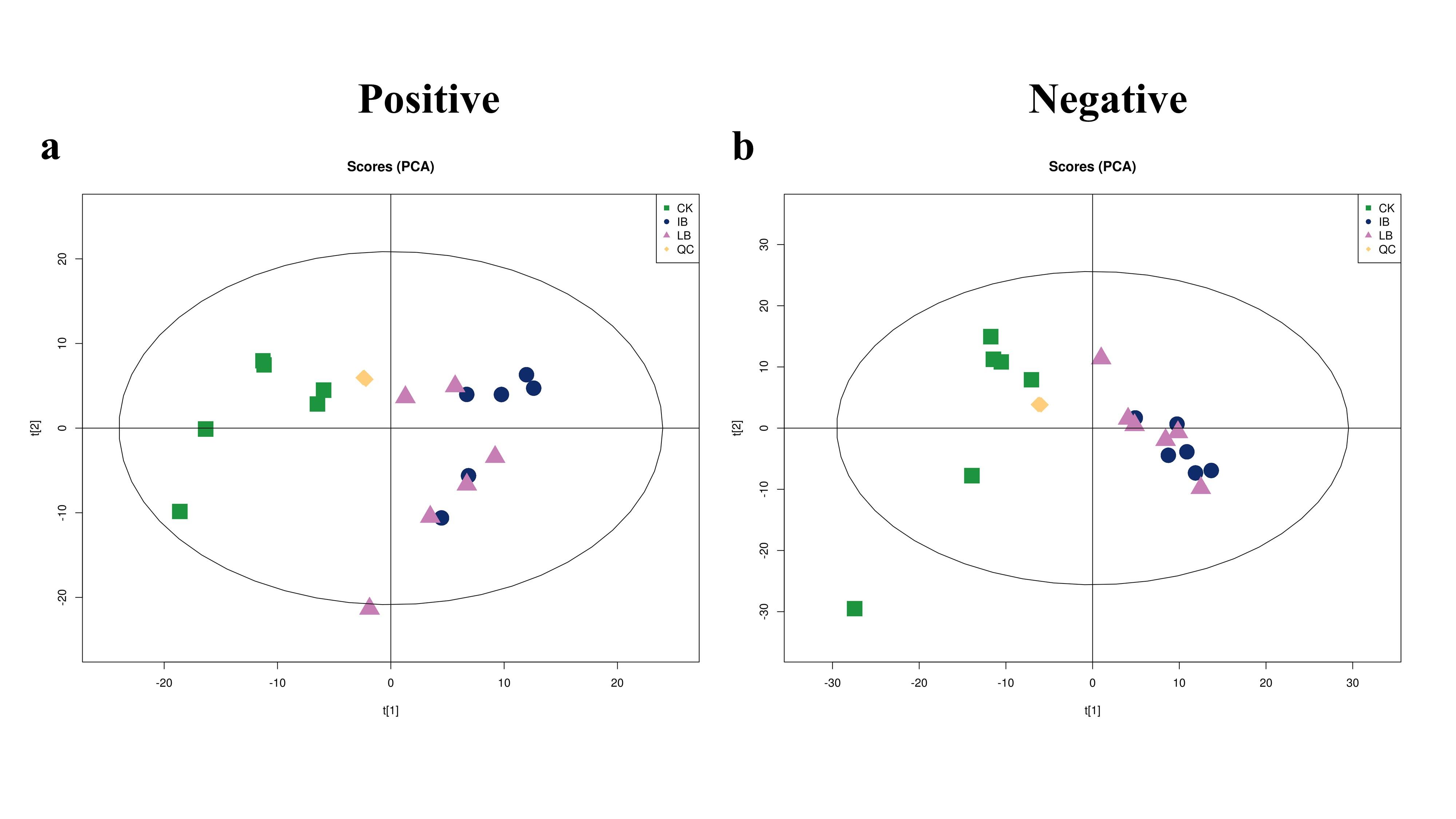

Supplement: Additional file : Fig. S2 — PCA analysis of fecal metabolomic samples with various treatments in positive and negative ion model. [file spectrum.00189-23-s0003.jpg]

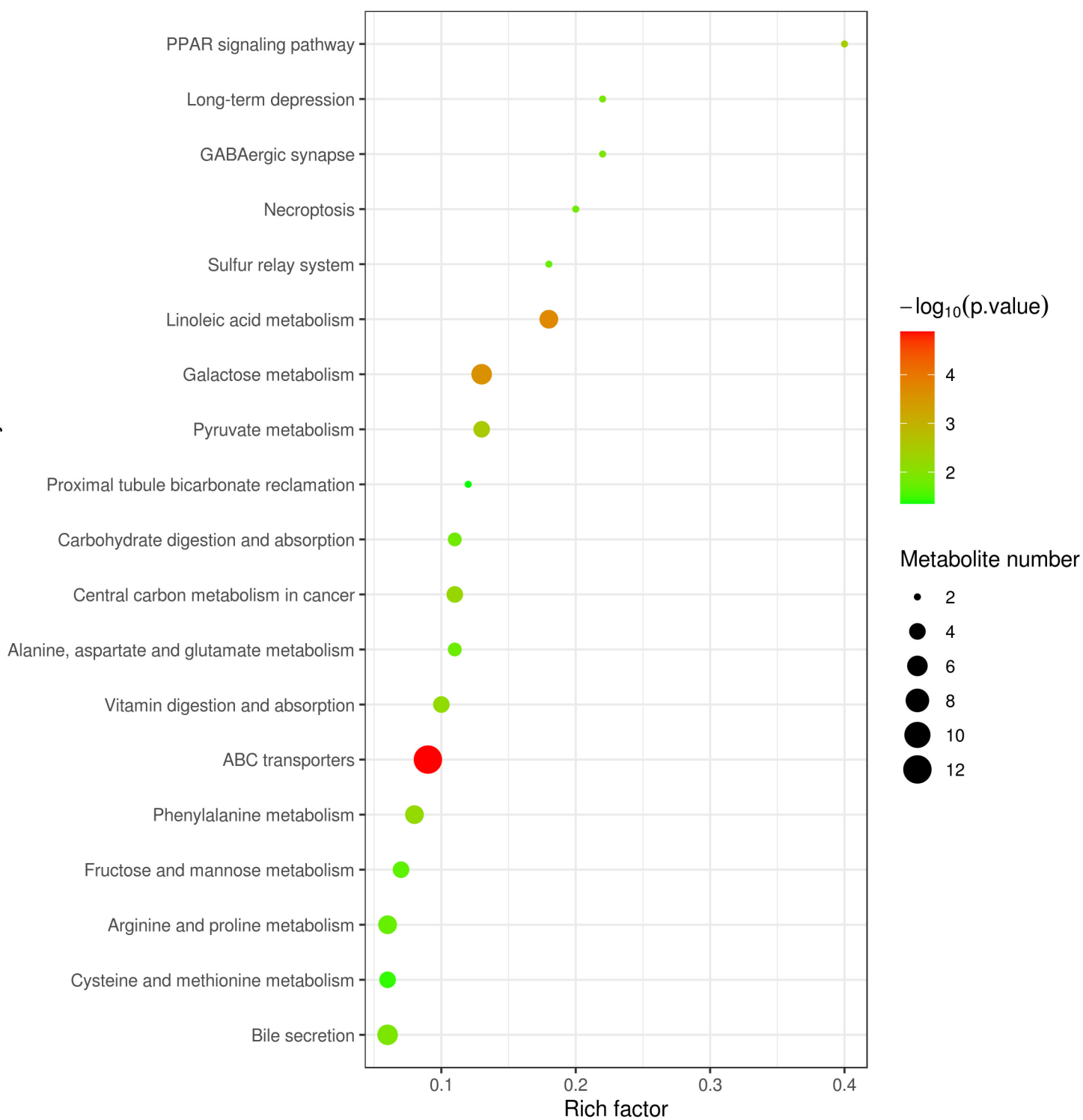

Supplement: Additional file : Fig. S3 — KEGG enrichment analysis in control_LB groups. [file spectrum.00189-23-s0004.pdf]

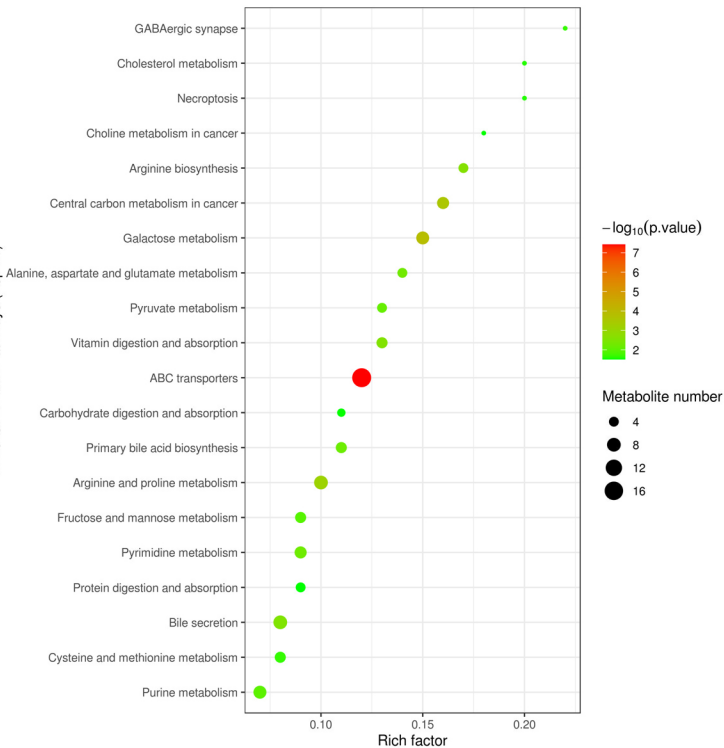

Supplement: Additional file : Fig. S4 — KEGG enrichment analysis in control_IB groups. [file spectrum.00189-23-s0005.pdf]

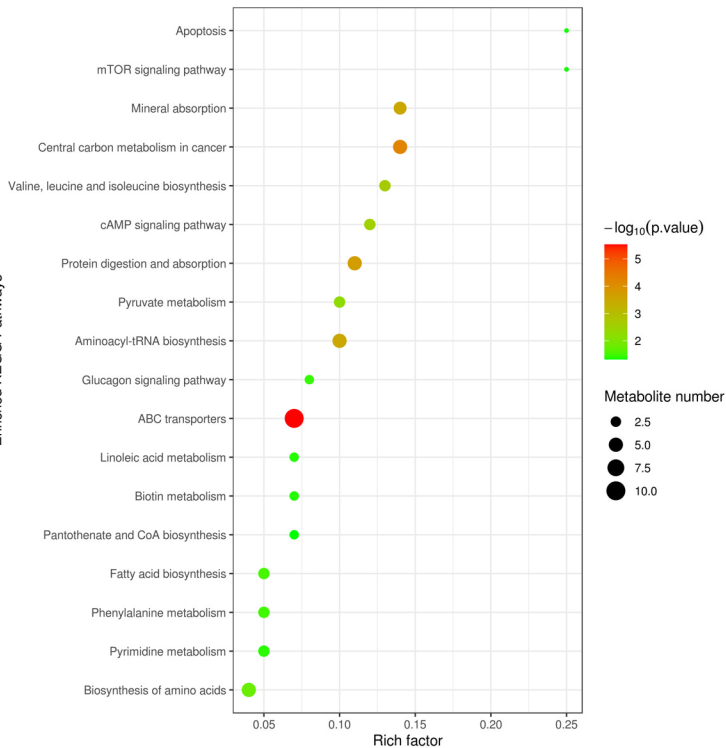

Supplement: Additional file : Fig. S5 — KEGG enrichment analysis in LB_IB groups. [file spectrum.00189-23-s0006.pdf]
