# Supplementary material for: The efficacy of prevention for colon cancer based on the microbiota therapy and the antitumor mechanisms with intervention of dietary Lactobacillus
Source: Microbiol Spectr. 2023 Sep 1;11(5):e00189-23. doi: 10.1128/spectrum.00189-23 (PMC10581183; doi:10.1128/spectrum.00189-23)
Supplement: Supplemental legends — Legends for supplemental figures and tables. [file spectrum.00189-23-s0007.docx]

**Supplementary Information**

**Additional file 1: Fig. S1.** Correlation map of QC samples ((a) in positive ion model and (b) in negative ion model)

**Additional file 2: Fig. S2a-b.** PCA analysis of fecal metabolomic samples with various treatments in positive and negative ion models.

**Additional file 3: Table S1-S3.** The metabolites with significant difference in various treatments.

**Additional file 4: Fig. S3.** KEGG enrichment analysis in control_LB groups.

**Additional file 5: Fig. S4.** KEGG enrichment analysis in control_IB groups.

**Additional file 6: Fig. S5.** KEGG enrichment analysis in LB_IB groups.

**Additional file 7: Table S4.** Differential metabolites between LB culture supernatant and IB suspension solution.
