# Supplementary material for: The efficacy of prevention for colon cancer based on the microbiota therapy and the antitumor mechanisms with intervention of dietary Lactobacillus
Source: Microbiol Spectr. 2023 Sep 1;11(5):e00189-23. doi: 10.1128/spectrum.00189-23 (PMC10581183; doi:10.1128/spectrum.00189-23)
Supplement: Additional file : Table S4 — Differential changed metabolites between LB culture supernatant and IB suspension solution. [file spectrum.00189-23-s0008.docx]

**Table S4. Differential changed metabolites between LB culture supernatant and IB suspension solution**

| **Name** | **LB1** | **LB2** | **LB3** | **IB1** | **IB2** | **IB3** |
| --- | --- | --- | --- | --- | --- | --- |
| L-histidinol | 3901875.2 | 3874629.5 | 4268292.7 | 8071950.1 | 9276012.3 | 8855239.3 |
| Spermidine | 157777957.7 | 162890510.0 | 169082262.9 | 212824513.6 | 352441253.1 | 55437553.1 |
| Spermine | 85498224.0 | 112871080.8 | 87384146.5 | 411989290.6 | 500657485.7 | 472153266.7 |
| 1-piperideine | 10580135.8 | 10898925.1 | 11663366.9 | 7570324.8 | 8724318.6 | 10658588.8 |
| Cadaverine | 13441220.3 | 21794236.2 | 21285070.6 | 6769465.1 | 7788637.1 | 7632120.1 |
| Hydroxylysine | 7028367.7 | 10474359.1 | 9979521.1 | 60188385.4 | 60848926.4 | 69896166.2 |
| (2e)-2,5-dichloro-4-oxo-2-hexenedioic acid | 1711723.1 | 1732487.7 | 2029279.3 | 9880519948.7 | 11594071218.1 | 10848173773.8 |
| D-ornithine | 241284823.1 | 230259017.3 | 262144468.9 | 920467758.7 | 562790643.8 | 1233750588.7 |
| Xanthurenic acid | 14145255.3 | 14036967.7 | 14932172.6 | 201000699.2 | 216388633.7 | 208131340.6 |
| 5-aminoimidazole ribotide | 133546992.2 | 127343601.0 | 134672460.1 | 134043204.2 | 152846624.4 | 147372092.0 |
| Choline | 425393379.1 | 422073013.3 | 458109176.1 | 3066390135.0 | 1164432479.3 | 3119215199.8 |
| O-phosphorylethanolamine | 7811154.2 | 7405167.3 | 7893771.5 | 19446459.1 | 27209591.6 | 22453869.0 |
| L-arginine | 45963780.2 | 45526717.8 | 48313733.6 | 82345621.5 | 86898931.3 | 80680511.1 |
| L-serine o-phosphate | 1515440.2 | 1802142.8 | 1627129.2 | 7488633.8 | 15417001.2 | 6359551.7 |
| N-methylethanolamine phosphate | 17748202.7 | 17731885.2 | 19568259.5 | 107924912.8 | 143714564.2 | 106161152.9 |
| Homocarnosine | 14040558.9 | 13194200.5 | 14867432.0 | 8107362.6 | 8217273.2 | 13818895.6 |
| Β-alanine | 97735696.9 | 99494234.8 | 59809481.7 | 611530435.7 | 612992818.3 | 596814252.3 |
| Gamma-aminobutyric acid | 813936451.6 | 832469994.5 | 754562424.5 | 3853945339.2 | 3592778888.4 | 3716314817.0 |
| Caffeine | 10489935.5 | 9067875.9 | 9403750.7 | 9091495.6 | 9222119.7 | 8827280.2 |
| Trigonelline | 2677710.4 | 3274017.7 | 2812166.3 | 16521840.1 | 6061891.1 | 37408214.8 |
| Creatine | 124836456.6 | 118780754.3 | 125796876.9 | 59977725.4 | 69749472.6 | 24879479.8 |
| Cytosine | 2648174.5 | 4416232.7 | 3834332.7 | 420653335.9 | 401954651.5 | 439929681.8 |
| L-histidine | 63271898.4 | 68113713.1 | 65637591.1 | 596058332.8 | 631669857.2 | 589336548.9 |
| Fructoselysine | 20425875.0 | 12182822.8 | 12205916.7 | 7757028.7 | 7802730.3 | 7871017.4 |
| L-lysine | 10368747.6 | 5852377.2 | 7199428.7 | 510403309.9 | 755389063.9 | 660392273.8 |
| (2s)-5-carbamimidamido-2-(2-oxo-1-azetidinyl)pentanoic acid | 15207989.3 | 14676330.9 | 15411730.7 | 29689922.5 | 26291564.0 | 27626767.8 |
| N-((4-amino-2-methyl-5-pyrimidinyl)methyl)formamide | 3775665.3 | 2750886.9 | 4508807.6 | 562052975.9 | 622447874.8 | 651873453.1 |
| Saccharopine | 38761325.4 | 33066471.1 | 35559998.6 | 50922341.9 | 50507599.3 | 56971971.2 |
| 2'-deoxycytidine | 3151852.4 | 2126121.4 | 2362273.8 | 526184839.6 | 454117291.0 | 471275450.7 |
| 2,4-diaminotoluene | 6492744.9 | 6370853.2 | 6635495.7 | 6350893.5 | 6388310.7 | 6444219.3 |
| Trans-5-o-(4-coumaroyl)-d-quinic acid | 1508368.8 | 1297045.8 | 1669542.3 | 2632071.9 | 2647579.1 | 2670750.0 |
| Caprolactam | 11883326.0 | 11509527.2 | 12441677.5 | 256508925.8 | 273601066.4 | 269298109.8 |
| 4-guanidinobutyric acid | 12222314.5 | 10876804.0 | 11734198.2 | 49351060.1 | 43548640.2 | 47942446.5 |
| Adenine | 702832622.1 | 602735674.3 | 617093803.5 | 23975644028.3 | 22131099746.4 | 22535111676.5 |
| Pyridoxine | 36570190.6 | 39692886.7 | 46425474.5 | 200708620.0 | 131983858.6 | 147013509.4 |
| (+)-allo-octopine | 3680792.2 | 3536538.4 | 4101175.0 | 10285913.1 | 13424368.9 | 9225962.6 |
| Guanine | 49016502.7 | 47948060.9 | 54021255.3 | 5198796769.9 | 5187673645.4 | 4300385279.9 |
| 6-acetamido-2-oxohexanoic acid | 2517962.2 | 1761161.3 | 2309544.0 | 8681308.5 | 8732455.5 | 22165588.6 |
| 2-aminoadipic acid | 31766905.9 | 16566956.0 | 18466883.6 | 117480084.2 | 200845397.0 | 165005264.1 |
| Dimethyl sulfoxide | 214814.8 | 227290.3 | 224901.2 | 113634015.2 | 56514812.3 | 292976098.8 |
| Pipecolate | 144374153.7 | 140041044.2 | 233257997.7 | 687081133.8 | 707033487.0 | 541466090.1 |
| 4-aminophenol | 5270612.6 | 4745345.0 | 5618935.6 | 19864345.2 | 18561652.1 | 16497763.5 |
| Lotaustralin | 40931965.7 | 40075965.4 | 41550128.8 | 8410268.7 | 9591225.7 | 8077763.8 |
| Pilocarpine | 52892137.2 | 56028566.4 | 57020621.5 | 9933310.0 | 11564038.1 | 8793236.4 |
| L-valine | 1025341929.2 | 1099097594.0 | 1721197444.8 | 1276235874.2 | 1225926775.1 | 1203197038.0 |
| Phosphoric acid | 18285913.7 | 31668681.8 | 47711116.7 | 18204118751.1 | 16301635978.7 | 17715508942.1 |
| 4-acetamidobutanoic acid | 29439664.0 | 37862743.1 | 26712915.1 | 26471897.8 | 26627860.5 | 243892159.5 |
| L-threonine | 110471895.6 | 131334666.4 | 89226258.9 | 708500881.3 | 867390809.9 | 735464256.3 |
| L-methionine | 250011087.4 | 297925713.1 | 193397713.7 | 11054207.3 | 13263669.7 | 21383852.4 |
| L-glutamic acid | 252798155.7 | 357916881.3 | 204922038.2 | 1411549779.9 | 2033614606.8 | 3551879574.9 |
| Acpc | 42688146.2 | 74784102.7 | 55735629.1 | 265054837.7 | 238663044.1 | 309254808.8 |
| L-(+)-aspartic acid | 13714038.8 | 12724522.0 | 25772477.7 | 88873691.6 | 90912814.4 | 91193172.4 |
| Uracil | 24268780.9 | 24564550.7 | 26671880.6 | 1777366536.9 | 1686295034.6 | 1013371740.6 |
| 2-amino-2,3,7-trideoxy-d-lyxo-hept-6-ulosonic acid | 3249166.5 | 3107375.9 | 3261821.8 | 18565034.1 | 5898579.8 | 18471052.2 |
| L-serine | 32585256.6 | 26951726.5 | 17861855.4 | 202450647.6 | 204235492.3 | 90459379.5 |
| Methionine sulfoxide | 20342568.3 | 18027411.2 | 19471155.2 | 31790696.1 | 63314016.9 | 56225525.4 |
| Adenosine-monophosphate | 24135162.7 | 19481764.0 | 21527609.8 | 420257745.6 | 398444000.5 | 399546035.9 |
| Cytidine 5'-monophosphate | 11839008.4 | 12560625.4 | 10146284.2 | 9311375.2 | 64557960.1 | 22567915.2 |
| Hypoxanthine | 72091799.6 | 90928897.1 | 77436143.5 | 124662859.9 | 87003892.6 | 89079975.3 |
| Nicotinamide | 3575161.2 | 4518935.8 | 3775474.3 | 5603055558.9 | 5267640464.1 | 5801290574.0 |
| Nicotinic acid | 40628450.4 | 42365723.8 | 40435466.5 | 1428986721.1 | 1416650163.0 | 1835856699.8 |
| 6185892 | 329453.1 | 301246.6 | 308799.6 | 115066689.4 | 93361726.2 | 273033246.9 |
| Adenosine cyclic 3',5'-monophosphate | 9619505.5 | 4575641.6 | 5699731.6 | 1543493.7 | 1569220.8 | 1534373.3 |
| Pyroglutamate | 1049504125.5 | 1034488961.7 | 689211494.6 | 1727722859.4 | 1220446633.5 | 414275400.5 |
| Acetophenone | 3444738001.8 | 2229934236.9 | 2019321055.6 | 174671861.2 | 132026758.2 | 88729796.3 |
| Trans-aconitic acid | 34359894.8 | 32621038.2 | 36130550.7 | 77023719.3 | 79284478.3 | 76904203.4 |
| Citric acid | 91249062.9 | 85712069.4 | 88130569.0 | 184720044.2 | 197753170.8 | 181860480.9 |
| Alpha-ketoglutaric acid | 28011872.2 | 26617301.0 | 25086721.1 | 69190116.6 | 72300054.1 | 72601816.7 |
| 2,5-furandicarboxylic acid | 53879407.2 | 57166580.7 | 51474542.9 | 111936662.4 | 141556227.0 | 146144654.9 |
| Xanthine | 34676714.0 | 17665154.8 | 25153765.7 | 276488233.3 | 279631342.9 | 299662357.8 |
| Tyramine | 547327986.2 | 459802539.8 | 588296878.0 | 62787215.3 | 74977698.9 | 79708087.3 |
| Ophthalmic acid | 19982301.3 | 14000055.5 | 14327526.9 | 2130033.9 | 11124457.3 | 2317412.5 |
| 3'-adenosine monophosphate (3'-amp) | 96874311.4 | 95938730.7 | 110573780.9 | 31525588.8 | 255499866.7 | 34917836.0 |
| Adenosine 2',3'-cyclic monophosphate | 30601894.2 | 23875645.5 | 31515732.5 | 3285599.8 | 3125035.6 | 3004021.5 |
| L-isoleucine | 852088276.3 | 2532292965.9 | 343194690.4 | 4748797491.8 | 2046726914.7 | 5270390341.2 |
| O-succinyl-l-homoserine | 2602764.5 | 2826987.8 | 3056836.8 | 211202504.6 | 247152107.6 | 203563176.4 |
| Thymine | 7844642.0 | 9401176.5 | 11665310.5 | 13082609.6 | 18237348.4 | 26224782.8 |
| Adenosine | 2051721.3 | 1714357.3 | 146991.9 | 5049422398.7 | 4438736837.3 | 5133802994.2 |
| Thymidine | 19704193.8 | 16112549.6 | 33276071.1 | 10876588.2 | 27575014.5 | 28616114.5 |
| 2-oxospirilloxanthin | 3686755.8 | 2829995.6 | 3251989.9 | 5803481.4 | 5642668.4 | 5343457.7 |
| Dihydroxyindole | 1052158.1 | 1202620.2 | 1364112.1 | 8732012.7 | 7644889.9 | 6398789.5 |
| 7-aminomethyl-7-deazaguanine | 3289826.0 | 3100496.0 | 3206536.9 | 4127540.7 | 4525882.8 | 3776027.4 |
| 1-methoxypyrene | 505551.0 | 914991.4 | 2194526.5 | 3469143.8 | 3485626.6 | 2826804.2 |
| Serotonin | 2882104.7 | 1639124.0 | 2701064.4 | 965758.6 | 3055476.1 | 1565514.5 |
| Hydroquinone | 9541067.3 | 6553352.9 | 10281524.8 | 19866614.6 | 28769431.2 | 17287194.9 |
| 4-methyl-5-thiazoleethanol | 4082904.1 | 3307681.5 | 3879888.8 | 79394126.0 | 82647166.1 | 81990755.5 |
| Anthranilic acid | 17927371.6 | 6693066.1 | 23672378.3 | 13087849.6 | 18697096.2 | 17826477.0 |
| L-phenylalanine | 1320767524.4 | 5428522020.7 | 1486267745.8 | 2926906272.7 | 3111676662.3 | 2831911514.7 |
| Cinnamic acid | 5113302435.3 | 5522383239.1 | 5328712058.9 | 2987970466.6 | 3150434796.7 | 2897721307.0 |
| Butyryl-l-homoserine lactone | 3760683.9 | 3191632.0 | 2618109.6 | 11717386.3 | 6206511.5 | 9672348.6 |
| Pyridoxamine | 115309565.7 | 175546487.6 | 121954722.6 | 41600800.3 | 16042921.0 | 37914695.0 |
| L-5-hydroxytryptophan | 8090348.4 | 13219206.2 | 8997973.1 | 23357375.3 | 30957581.6 | 43296065.8 |
| Dihydrozeatin-o-glucoside | 1032150.8 | 1796944.7 | 310759.4 | 2560267.8 | 1903074.6 | 2127840.1 |
| Atropine | 11842265.1 | 5636889.6 | 11957967.8 | 560412.5 | 586467.6 | 640593.4 |
| Tryptamine | 3198426.2 | 2728422.8 | 5424779.6 | 708005.3 | 1753038.4 | 718160.3 |
| Benzene | 93488.4 | 1613525.6 | 1473866.8 | 1841887.1 | 1675046.1 | 1683156.7 |
| L-glutamine | 2422383.5 | 2078266.1 | 2743988.9 | 20886638.9 | 28526675.6 | 23986597.3 |
| L-kynurenine | 709357.1 | 5042847.9 | 5509744.4 | 15422976.3 | 13837062.7 | 14154443.1 |
| Porphobilinogen | 104189.3 | 16037852.6 | 14511635.7 | 97747086.7 | 93196029.9 | 105487849.1 |
| Deacetylvindoline | 3093639.0 | 873026.8 | 2413139.3 | 487025.7 | 586819.1 | 504739.7 |
| (+/-)-pantetheine | 16005534.8 | 13818444.6 | 6110818.0 | 1066586.4 | 863744.8 | 851424.8 |
| Trans-zeatin | 21949646.8 | 5760270.5 | 4478640.3 | 277099.8 | 324642.4 | 279234.1 |
| Paspalinine | 1483484.2 | 788129.6 | 1219186.5 | 359355.9 | 421011.3 | 369244.6 |
| (-)-physostigmine | 5789905.9 | 6104244.1 | 36885349.3 | 6055388.7 | 6061768.0 | 5720841.8 |
| 2,5-diamino-6-hydroxy-4-(5-phosphoribosylamino)pyrimidine | 2625423.2 | 2343124.0 | 2516201.6 | 433045.2 | 507343.7 | 444961.7 |
| Styrene | 148194.2 | 141134.2 | 6512292.7 | 77364069.8 | 96749938.8 | 83012856.1 |
| Magnoflorine | 587194.5 | 1460455.3 | 1464759.2 | 481958.3 | 515462.7 | 497221.0 |
| Spectinomycin | 1343681.5 | 673374.3 | 10335995.5 | 2069482.7 | 3938380.6 | 2150026.6 |
| (+/-)-nicotine | 1126575.2 | 192637.3 | 416996.5 | 1696340.4 | 4307502.2 | 1122586.8 |
| Ftc | 4037197.8 | 6593458.7 | 6588438.6 | 2618972.1 | 4451677.7 | 2036206.8 |
| Indole | 24188002.1 | 208948.6 | 657989.4 | 27784933.4 | 39774420.6 | 35415506.4 |
| 10-deacetylbaccatin iii | 8858226.0 | 3742866.9 | 8925273.4 | 4267338.8 | 4590855.5 | 4557216.7 |
| Pyrrole-2-carboxylic acid | 258493.2 | 255395.5 | 264145.2 | 141476886.0 | 95714610.4 | 436502024.9 |
| 6-hydroxypseudooxynicotine | 43475002.5 | 20658897.7 | 28564467.5 | 146243340.5 | 165241831.3 | 155964172.1 |
| Sepiapterin | 679994.2 | 222204.7 | 261920.7 | 156379467.4 | 177229311.2 | 126723225.4 |
| N~6~-(5-oxo-d-isoleucyl)-l-lysine | 9833791.5 | 8573991.4 | 9821509.1 | 1496293.6 | 1277791.4 | 1581817.3 |
| Biotin | 10027886.3 | 9361503.2 | 10221586.0 | 5671578.8 | 5750362.3 | 6478252.2 |
| Biotin l-sulfoxide | 16342390.4 | 7965252.0 | 11850871.0 | 28489378.0 | 31229253.7 | 30278693.2 |
| 5'-methylthioadenosine | 155982.5 | 510951.0 | 1044213.5 | 6669619700.3 | 6846161556.8 | 5867900730.8 |
| D-(-)-morphine | 1153960.6 | 2585653.0 | 1116378.2 | 1994952.1 | 2898145.5 | 1517276.7 |
| Pyrene | 1021803.0 | 1054194.1 | 1209185.7 | 781472.8 | 521176.4 | 644757.2 |
| Linatine | 13098780.5 | 11680514.7 | 13529476.6 | 8133248.3 | 3427369.5 | 7255159.0 |
| Salidroside | 1862942.5 | 1795373.4 | 1857772.2 | 3194171.6 | 3206041.3 | 3229121.9 |
| Hippurate | 1964481.1 | 1704291.4 | 2268881.4 | 5593024.2 | 8667709.6 | 7121368.0 |
| Vincristine | 1146065.8 | 1197792.6 | 1214547.5 | 939630.1 | 723865.9 | 795491.8 |
| Leukotriene c4 | 540659.8 | 609957.0 | 1124439.1 | 1082639.3 | 734589.2 | 899704.9 |
| 3,4-dihydroxyphenylglycol | 2220819.2 | 2289271.7 | 2114241.9 | 898126.2 | 2733254.9 | 746369.1 |
| L-tryptophan | 13514771.7 | 14342678.6 | 15161527.6 | 3559309.0 | 6078604.6 | 5037015.5 |
| 6-hydroxymelatonin | 4518562.2 | 5106666.9 | 5134133.2 | 3476109.4 | 3092779.7 | 3203321.9 |
| 3,4-dihydroxyphenylacetic acid | 1771679.0 | 1547422.3 | 764469.4 | 739948.5 | 1477495.9 | 2415389.2 |
| Viomycin | 792312.2 | 883014.2 | 849944.9 | 526193.3 | 520548.6 | 519077.2 |
| 3-oxo-c12-hsl | 198105.2 | 772274.4 | 708877.4 | 436980.1 | 2205220.0 | 1717900.7 |
| 4-aminobenzoate | 208219569.1 | 223784137.2 | 210397962.8 | 151772420.8 | 164994035.3 | 154340697.5 |
| Î³-oxo-3-pyridinebutanal | 1590033.0 | 1502414.5 | 1811174.9 | 2231136.9 | 2280503.5 | 2279440.6 |
| Biocytin | 3366302.3 | 3922917.5 | 3577582.0 | 997591.5 | 946529.4 | 941930.5 |
| Nebramycin 5' | 349835.5 | 644057.5 | 663923.3 | 429001.2 | 426582.6 | 405503.5 |
| N-acetylserotonin | 9257863.2 | 10103707.6 | 9934612.5 | 20099333.7 | 18494368.3 | 18553339.5 |
| Lividomycin | 500180.3 | 578911.1 | 368827.0 | 243172.5 | 271519.7 | 230076.6 |
| L-pyrrolysine | 2257244.7 | 1824022.3 | 1461572.3 | 570259.9 | 555901.8 | 580151.3 |
| Lolitrem b | 3826973.3 | 3976238.7 | 3907914.5 | 577636.3 | 624358.2 | 547316.9 |
| Diacetyl | 79454.1 | 80438.8 | 86948.1 | 16874990.7 | 15701240.2 | 145540566.8 |
| Tyrosol | 90467.3 | 91588.4 | 99000.0 | 29874724.1 | 12778245.4 | 67919695.5 |
| Indole-3-acetaldehyde | 3149753.8 | 3297023.5 | 3284635.6 | 73815310.0 | 87521626.1 | 72644274.7 |
| 3-methoxytyramine | 248567.0 | 261633.6 | 273505.5 | 5248538.7 | 4096487.0 | 57154767.3 |
| N6-(delta2-isopentenyl)-adenine | 6288479.8 | 7061610.9 | 1405734.9 | 337382631.6 | 349615745.6 | 335487236.7 |
| (1s,6s)-6-aminooctahydro-1-indolizinyl acetate | 4135027.3 | 3991210.4 | 4236123.3 | 1263198.7 | 932880.2 | 1103423.0 |
| N-acetyl-l-phenylalanine | 2096602.5 | 2059734.5 | 2049650.3 | 17670974.6 | 36747017.8 | 29802483.6 |
| Mycaminosyltylonolide | 2458627.5 | 2707746.2 | 2764074.1 | 933269.2 | 991315.4 | 984589.0 |
| Melatonin | 138370.3 | 438141.2 | 146747.6 | 8991284.9 | 205598184.2 | 17089802.2 |
| Coumarin | 5504068.8 | 5581663.0 | 7879876.1 | 1594099078.7 | 1394554833.5 | 1379948021.5 |
| (2e,6e)-farnesyl monophosphate | 13369603.2 | 13017324.6 | 13389030.6 | 14769563.2 | 16827732.7 | 15637380.4 |
| Isobutyric acid | 25243069.3 | 24875251.8 | 28552169.3 | 266118232.9 | 313442897.4 | 316461712.0 |
| Lumichrome | 960299.5 | 1062392.1 | 838998.3 | 74603929.4 | 81056228.2 | 81029544.1 |
| 5-ribosylparomamine | 6442082.4 | 6815804.6 | 7209038.9 | 6696933.5 | 7095078.9 | 6112295.7 |
| Paromomycin | 3251737.9 | 5729638.0 | 6168015.8 | 30734815.0 | 36970236.0 | 28655204.9 |
| Mycinamicin ii | 3514036.3 | 2979748.1 | 3672006.6 | 1664927.9 | 1979433.9 | 1839505.3 |
| Nylon cyclic dimer | 28340260.5 | 32634707.4 | 30244809.8 | 2581882.3 | 2466792.9 | 2444283.5 |
| Senecionine | 684959.7 | 629625.1 | 604287.8 | 1479140.8 | 1862506.4 | 1162066.7 |
| 5-methoxyindoleacetic acid | 207523.7 | 308005.5 | 244663.9 | 233229684.6 | 158459993.0 | 166367891.8 |
| Actinorhodin | 1227849.0 | 1342288.2 | 1126994.5 | 1775627.2 | 1871907.7 | 5765672.5 |
| Naphthalene | 27416.8 | 28732.6 | 28456.3 | 64129287.4 | 58246397.7 | 71599565.3 |
| D-(+)-camphor | 2805210.9 | 2911755.6 | 2883553.1 | 11675863.7 | 19433563.0 | 20303411.3 |
| (e)-p-coumaric acid | 34344.1 | 35928.4 | 35399.4 | 74616760.7 | 71897982.8 | 62029850.5 |
| 2-amino-1,3,4-octadecanetriol | 42583496.2 | 45436182.2 | 50760821.9 | 6428837275.0 | 7476704547.9 | 7016094073.2 |
| 12-oxo phytodienoic acid | 5243156.2 | 11980553.4 | 6298340.0 | 17920879.6 | 15151531.7 | 28382644.6 |
| P-xylene | 110828.3 | 96107.2 | 109543.8 | 270723503.0 | 292633006.3 | 264529431.8 |
| Cuminaldehyde | 208282.1 | 181614.1 | 205877.1 | 4253350322.4 | 4418999236.9 | 3749249821.7 |
| Trans-cinnamaldehyde | 42693.6 | 80291.1 | 88605.7 | 241514206.8 | 263379681.3 | 246804354.6 |
| Cinnamyl alcohol | 175291.2 | 101014.8 | 103582.9 | 210253301.0 | 236278667.3 | 207129345.7 |
| 13(s)-hpotre | 1803371.5 | 1528478.7 | 1368599.7 | 6534737.0 | 5241658.6 | 5328677.2 |
| Gamma-linolenic acid | 334366638.9 | 336225249.3 | 102928003.7 | 26664920.4 | 18181054.2 | 15984321.2 |
| Arachidonic acid | 22954137.1 | 20424698.7 | 20030145.1 | 4127283.5 | 2789870.9 | 3481929.8 |
| Istamycin ao | 8245496.3 | 4196331.9 | 9668539.5 | 1885205.0 | 1941012.8 | 1991674.8 |
| Palmitoleic acid | 4983340.4 | 5401884.7 | 4460678.8 | 147728749.6 | 138570888.6 | 158680724.8 |
| Oleate | 426239022.9 | 520843877.4 | 461936543.5 | 1093013897.1 | 1019472564.4 | 1078118731.5 |
| Citral | 10273232.7 | 12013080.0 | 10345600.9 | 21041293.7 | 22883935.9 | 26861396.5 |
| (3e,5s,6s,7s,9r,11e,13e,15r,16r)-16-ethyl-15-(hydroxymethyl)-5,7,9-trimethyl-2,10-dioxooxacyclohexadeca-3,11,13-trien-6-yl 3,4,6-trideoxy-3-(dimethylamino)-beta-d-xylo-hexopyranoside | 2806098.9 | 2552000.3 | 2466079.3 | 3091380.8 | 3268873.1 | 3154946.5 |
| Tetralin | 3227675.2 | 1693843.0 | 1788264.9 | 62105003.9 | 84495529.3 | 71638412.2 |
| 5-o-mycaminosyprotylonolide | 1299888.7 | 1459792.9 | 1742414.8 | 2151766.3 | 2275310.4 | 2196011.5 |
| (-)-lupinine | 464198.3 | 435902.5 | 440224.8 | 67059876.9 | 77118539.7 | 106899120.3 |
| 8z,11z,14z-eicosatrienoic acid | 374861426.9 | 344674159.4 | 395623817.5 | 86499949.8 | 106909066.0 | 89282548.1 |
| Glycochenodeoxycholate | 193169255.4 | 209970171.4 | 213978117.0 | 17451854.2 | 26234305.3 | 18285496.1 |
| Citrate | 212383917.6 | 194168684.9 | 187253425.4 | 8294443028.6 | 9358190777.0 | 8335449888.3 |
| D-glucose 6-phosphate | 1679264.3 | 1536189.4 | 1550109.5 | 9320192.4 | 5078210.1 | 9590015.9 |
| Sulfuric acid | 61802768.1 | 56115116.6 | 55494211.1 | 974801824.3 | 658827468.1 | 1097863757.5 |
| Trans-aconitic acid | 21928138.2 | 21729441.8 | 20725285.8 | 117744009.8 | 136573373.2 | 124727269.3 |
| Oxoglutaric acid | 506679878.0 | 551357156.1 | 532755314.7 | 8190293716.4 | 9231784970.4 | 8221791278.0 |
| Phosphoric acid | 35497918.7 | 33028289.5 | 32630260.7 | 43917397444.2 | 31002044536.6 | 34758410426.4 |
| Pyruvic acid | 6280788.9 | 6857187.9 | 8587164.2 | 285505494.4 | 267782868.7 | 280192685.5 |
| 2-oxobutyric acid | 23701689.6 | 22559774.3 | 21119787.6 | 445295174.7 | 477566752.5 | 429678491.9 |
| N-acetyl-l-aspartic acid | 2863379.4 | 2941583.9 | 2968658.7 | 37309400.5 | 39150709.8 | 38690583.7 |
| D-aspartate | 29990831.3 | 30390130.7 | 28666892.7 | 814645243.9 | 930198166.1 | 641647698.4 |
| Gluconic acid | 13528034.7 | 4782708.4 | 7256805.5 | 34379507.0 | 18919501.1 | 74148681.3 |
| 4129939 | 8177322.0 | 5441077.2 | 8100427.2 | 29353803.4 | 22320130.2 | 13656463.5 |
| L-asparagine | 1448117.5 | 639790.1 | 908423.1 | 5298009.8 | 4737978.1 | 4930680.7 |
| D-(-)-fructose | 48895593.4 | 99981089.6 | 121942523.7 | 63553764.1 | 62346031.5 | 79295009.0 |
| D-(-)-ribose | 10367006.9 | 9222943.5 | 10727251.1 | 165992773.2 | 134660482.3 | 249503615.2 |
| L-citrulline | 3367413.5 | 3590117.2 | 3696019.2 | 188375495.8 | 204250222.3 | 270435386.3 |
| L-threonine | 37888599.7 | 49299299.4 | 42557091.2 | 607812493.5 | 809976853.4 | 658079575.6 |
| Acrylic acid | 5410137.1 | 3711071.0 | 3256185.7 | 18284936.3 | 15835731.0 | 16387863.9 |
| 3-ureidopropionic acid | 23762237.4 | 24096327.5 | 32148911.9 | 109347648.0 | 83353169.8 | 109268414.9 |
| L-histidine | 40342470.1 | 45125510.4 | 42927053.2 | 857715597.2 | 964396430.2 | 979599937.8 |
| N-acetylneuraminate | 47809742.2 | 45916089.4 | 45158709.9 | 8843227.9 | 8470869.7 | 10444095.8 |
| L-glutamic acid | 383159719.5 | 414523589.2 | 396923005.3 | 2231408052.1 | 1726295401.0 | 2267551838.5 |
| Carnosine | 5709074.6 | 6383524.4 | 6552867.9 | 5595859.6 | 6357751.6 | 5730330.1 |
| 2-aminoadipic acid | 23403015.0 | 25938999.7 | 23546277.2 | 259840947.2 | 255285826.2 | 262362797.9 |
| Caffeine | 2296118.0 | 2340233.0 | 2369488.7 | 11733289.5 | 11141754.9 | 13192158.8 |
| Cytidine 5′-monophosphate | 6408128.6 | 6970856.9 | 6430842.2 | 52980322.8 | 50425932.4 | 47965747.0 |
| Uridine 5'-monophosphate | 17112335.3 | 17496706.5 | 15984940.6 | 239542127.6 | 303911610.8 | 290615513.3 |
| 2-keto-glutaramic acid | 1339151.8 | 1396174.3 | 1385363.4 | 8505946.0 | 7675851.0 | 8786711.9 |
| 2'-deoxycytidine | 1002617.0 | 1129595.0 | 1098330.9 | 198685657.8 | 159951643.1 | 161550913.5 |
| Linatine | 1510484.1 | 1540943.7 | 1988029.2 | 13214441.7 | 11924844.8 | 13526897.4 |
| Guanosine monophosphate (gmp) | 12782247.7 | 13070066.7 | 13646062.5 | 251888209.1 | 265582320.7 | 255587284.2 |
| Guanine | 586981.2 | 850209.6 | 791607.6 | 677277889.5 | 708453369.1 | 614010312.3 |
| L-proline | 5221520.5 | 5343023.4 | 4860923.7 | 77756161.0 | 85700373.3 | 79277567.5 |
| Uracil | 10465035.7 | 11055705.0 | 10158599.5 | 1428808701.9 | 1254299281.3 | 1281512572.4 |
| Δ-gluconic acid δ-lactone | 10433784.4 | 1772083.7 | 4526519.2 | 11557122.5 | 10974469.3 | 15387809.2 |
| Xanthine | 13460727.6 | 13264306.3 | 13026617.7 | 508243240.1 | 464670466.3 | 489622130.1 |
| Β-alanine | 6702949.9 | 6075730.7 | 6322416.5 | 420273256.6 | 364469013.6 | 352340578.6 |
| Deoxyuridine monophosphate | 17282927.2 | 13360456.4 | 19454200.1 | 15299668.9 | 14528335.0 | 17201967.2 |
| L-(+)-lactic acid | 5853064190.9 | 5969812876.4 | 4796669941.1 | 1379021478.1 | 944869500.4 | 17515982768.7 |
| 4-oxoproline | 181486784.9 | 181578668.3 | 164947414.3 | 305757898.4 | 401097371.2 | 283708841.2 |
| 2',3'-cyclic ump | 4461778.0 | 5070957.5 | 5823703.7 | 2607524.2 | 2139636.9 | 2353076.1 |
| Cis-aconitic acid | 13829899.3 | 16030883.0 | 14712955.2 | 1509685.1 | 1257509.7 | 1413928.6 |
| . | 6916050.8 | 7696457.1 | 7546496.6 | 1420802.3 | 1410703.4 | 1442191.9 |
| Succinate | 297381332.5 | 297329219.0 | 278050568.0 | 22566225.3 | 28591467.2 | 26618711.6 |
| L-(-)-methionine | 29147486.8 | 27445603.5 | 28583126.2 | 12913910.4 | 17912883.0 | 22309282.4 |
| Hypoxanthine | 3755222.4 | 4358918.5 | 3708998.7 | 23647829.3 | 28278400.2 | 19390384.7 |
| Ophthalmic acid | 1964096.8 | 1842726.4 | 2171153.0 | 1555020.9 | 1159061.9 | 1211364.3 |
| 5-aminovaleric acid | 75129898.0 | 76642980.1 | 74960623.5 | 519900285.3 | 28458119.6 | 24757096.7 |
| Glutaric acid | 2684045.6 | 2117255.3 | 2768368.9 | 7595361.6 | 6925119.6 | 9201428.0 |
| Thymidine | 4371416.9 | 2637141.8 | 3435420.8 | 3580783.3 | 6336561.5 | 5219290.2 |
| 4-methylene-2-oxoglutarate | 1477345.9 | 1632192.0 | 1563137.7 | 645244.9 | 677100.0 | 655902.1 |
| 3,4-dihydroxybenzoate | 3006214.9 | 2904376.7 | 2853568.1 | 609674.4 | 3431185.6 | 2499073.5 |
| Salicylic acid | 1386561.3 | 1897642.5 | 1720775.9 | 1953313.9 | 1309297.0 | 1046506.3 |
| Phthalic acid | 802488.9 | 901017.0 | 721375.7 | 508929.7 | 423336.1 | 434478.4 |
| Cgmp | 11862358.6 | 9709164.0 | 9356710.8 | 1342861.5 | 3282529.9 | 2886985.1 |
| Vanillic acid | 1970041.3 | 2005716.0 | 2083495.3 | 1399731.4 | 1542496.0 | 592948.7 |
| (z)-2-butene-1,2,3-tricarboxylic acid | 3411689.4 | 3391155.9 | 3877094.9 | 667382.3 | 652728.1 | 700034.3 |
| Adenosine 5'-monophosphate | 9113527.7 | 9997704.2 | 8739852.5 | 807877.0 | 2538798.6 | 2677368.8 |
| Fmet | 6796338.5 | 8343663.9 | 9504234.3 | 22031706.2 | 10896563.6 | 20573188.6 |
| Pantothenic acid | 13089869.0 | 19185611.5 | 18661784.2 | 1042489.4 | 991842.9 | 1058209.5 |
| Vanillyl mandelic acid | 370408.0 | 488792.1 | 373020.0 | 512704.5 | 503516.1 | 503685.5 |
| Adenine | 121397231.9 | 142670471.5 | 178685454.6 | 8859685229.6 | 8293801306.0 | 9150359767.1 |
| Porphobilinogen | 14648548.3 | 8129583.4 | 14299969.6 | 4123558.9 | 4592397.2 | 5299411.2 |
| 4-aminobenzoate | 11259463.6 | 11462622.5 | 10309433.2 | 6615469.9 | 8097213.0 | 5897776.9 |
| Stearic acid | 19629238.1 | 20675535.9 | 19557760.2 | 402017250.1 | 441538807.3 | 344719194.2 |
| (1s)-1-carboxy-n,n,n-trimethyl-2-(2-thioxo-2,3-dihydro-1h-imidazol-4-yl)ethanaminium | 3349064.2 | 3444370.6 | 3337778.6 | 738511885.0 | 699433400.9 | 967423319.0 |
| 3'-adenosine monophosphate (3'-amp) | 2427496.3 | 2441911.0 | 2425218.4 | 5470851.4 | 6991984.2 | 721361.7 |
| Adenosine 3'5'-cyclic monophosphate | 4156856.4 | 3069203.5 | 2940962.3 | 5100361.7 | 3036194.8 | 5045840.3 |
| L-dopa | 4731487.9 | 4720139.0 | 5914878.5 | 1621554.7 | 2299380.7 | 1596791.0 |
| Mhpg | 641585.0 | 78920.3 | 796817.8 | 104687787.2 | 97087493.9 | 358304248.1 |
| Acetophenone | 11635152.0 | 11061591.8 | 11038930.8 | 56389241.3 | 49822539.5 | 51234354.6 |
| 3-phenyllactic acid | 641528811.6 | 650013952.2 | 659830564.4 | 3341215435.6 | 3147438907.3 | 3016653218.4 |
| Indole | 695940.8 | 1056539.0 | 780701.4 | 2166430.7 | 1199012.4 | 1321302.1 |
| 6-acetamido-2-oxohexanoic acid | 2761874.8 | 2641621.0 | 2697252.1 | 8421237.2 | 2431402.9 | 7097267.9 |
| (e)-p-coumaric acid | 631849.2 | 640429.0 | 744967.5 | 2308853.2 | 818531.1 | 1507596.4 |
| Leukotriene c4 | 1730708.7 | 1239712.7 | 954078.3 | 920143.3 | 939345.7 | 928240.5 |
| N-acetyl-l-phenylalanine | 7655189.3 | 7551076.3 | 7147322.0 | 30113071.1 | 55544314.2 | 57265193.5 |
| 4-hydroxyphenylacetic acid | 1183600.9 | 1191492.9 | 1293342.4 | 2564722.9 | 2691208.0 | 2586335.2 |
| 4-hydroxybenzaldehyde | 5338973.2 | 5198604.2 | 5135370.8 | 87461135.8 | 70516956.5 | 126102331.7 |
| (+/-)-pantetheine | 744764.6 | 719010.0 | 653201.7 | 752244.2 | 804036.4 | 768584.5 |
| L-tryptophan | 124472365.9 | 148548988.7 | 130900240.0 | 255549089.2 | 290354247.6 | 279605699.6 |
| Indole-3-acetic acid | 695557.6 | 715929.9 | 698107.4 | 71732099.7 | 76227880.7 | 74910979.2 |
| Protoporphyrin ix | 1197629.4 | 1283265.7 | 1234649.1 | 905865.3 | 1010157.8 | 998104.4 |
| Indole-3-acetaldehyde | 422595.8 | 552199.4 | 482868.6 | 1918068.7 | 1242445.2 | 2708352.6 |
| Β-muricholic acid | 1694270.5 | 1714547.0 | 1692682.3 | 4630527.9 | 3739501.1 | 3948567.6 |
| 16-hydroxyhexadecanoic acid | 1524671.8 | 1625235.3 | 1549256.5 | 5571161.9 | 86149342.5 | 94205599.7 |
| Linoleic acid | 139282095.0 | 134710465.0 | 142512946.8 | 323580137.7 | 222736815.8 | 287922996.8 |
| Palmitic acid | 7393290.6 | 8508573.1 | 8394054.3 | 926294726.8 | 869633815.4 | 889027458.2 |
